# Stepwise optimization of tumor-targeted dual-action platinum(iv)-gemcitabine prodrugs†

**DOI:** 10.1039/d3qi02032k

**Published:** 2023-12-06

**Authors:** Alexander Kastner, Theresa Mendrina, Tomer Babu, Subhendu Karmakar, Isabella Poetsch, Walter Berger, Bernhard K. Keppler, Dan Gibson, Petra Heffeter, Christian R. Kowol

**Affiliations:** a University of Vienna, Faculty of Chemistry, Institute of Inorganic Chemistry Waehringer Str. 42 1090 Vienna Austria christian.kowol@univie.ac.at; b Center of Cancer Research and Comprehensive Cancer Center, Medical University of Vienna Borschkegasse 8a 1090 Vienna Austria petra.heffeter@meduniwien.ac.at; c Institute for Drug Research, School of Pharmacy, The Hebrew University of Jerusalem 9112102 Jerusalem Israel dang@ekmd.huji.ac.il; d Research Cluster “Translational Cancer Therapy Research” 1090 Vienna Austria; e University of Vienna, Vienna Doctoral School in Chemistry (DoSChem) Waehringer Str. 42 1090 Vienna Austria

## Abstract

While platinum-based chemotherapeutic agents have established themselves as indispensable components of anticancer therapy, they are accompanied by a variety of side effects and the rapid occurrence of drug resistance. A promising strategy to address these challenges is the use of platinum(iv) prodrugs, which remain inert until they reach the tumor tissue, thereby mitigating detrimental effects on healthy cells. Typically, platinum drugs are part of combination therapy settings. Consequently, a very elegant strategy is the development of platinum(iv) prodrugs bearing a second, clinically relevant therapeutic in axial position. In the present study, we focused on gemcitabine as an approved antimetabolite, which is highly synergistic with platinum drugs. In addition, to increase plasma half-life and facilitate tumor-specific accumulation, an albumin-binding maleimide moiety was attached. Our investigations revealed that maleimide-cisplatin(iv)-gemcitabine complexes cannot carry sufficient amounts of gemcitabine to induce a significant effect *in vivo*. Consequently, we designed a carboplatin(iv) analog, that can be applied at much higher doses. Remarkably, this novel analog demonstrated impressive *in vivo* results, characterized by significant improvements in overall survival. Notably, these encouraging results could also be transferred to an *in vivo* xenograft model with acquired gemcitabine resistance, indicating the high potential of this approach.

## Abstract 2

An albumin-binding cisplatin(iv)-gemcitabine complex was step-by-step improved based on stability, reduction, albumin-binding and *in vivo* data yielding a carboplatin(iv) complex with distinctly improved overall survival and anticancer activity.

## Graphical abstract

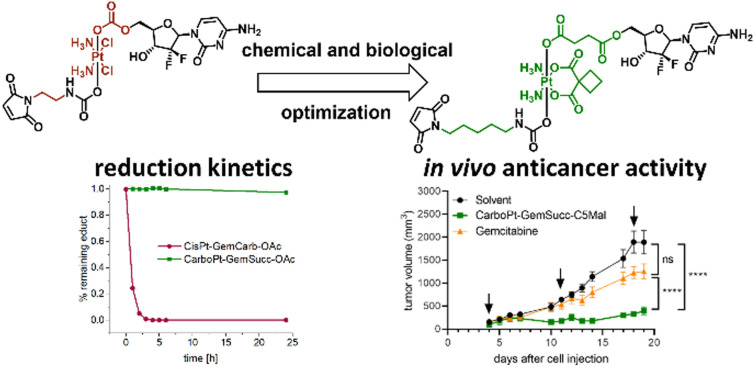

## Introduction

Cancer has become the second leading cause of death worldwide, resulting in approximately 9.6 million fatalities in 2018.^1^ Consequently, there have been tremendous efforts to discover new anticancer agents. Platinum complexes are a particularly effective class of compounds, which have become a standard component in many treatment schemes. Three platinum(ii) compounds, namely cisplatin, carboplatin, and oxaliplatin, have been approved for clinical use worldwide since the discovery of the anticancer activity of cisplatin by Barnett Rosenberg in 1965. Additionally, five more platinum(ii) compounds are clinically used in select Asian countries.^2^ These complexes are administered intravenously and enter the cells either through passive diffusion or active transport. After aquation and loss of the labile ligands, the platinum binds to DNA bases, specifically adenine and guanine, causing DNA damage and ultimately apoptosis.^3^ Unfortunately, this process also occurs in healthy tissue, leading to a variety of side effects, ranging from nausea and hair loss to severe nephro- and neurotoxicity.^4^

To address this issue, research has shifted towards platinum(iv) complexes, which are kinetically more inert.^5,6^ These compounds are considered prodrugs, as they only become activated (*i.e.*, reduced) to their active platinum(ii) counterpart in the tumor tissue.^7^ Unfortunately, only a few have entered clinical trials (including iproplatin, tetraplatin, and satraplatin), and none of them have yet been approved for clinical use, indicating that further improvements are necessary for this compound class.^5^ Octahedral d^6^-lowspin platinum(iv) further provides two additional ligands compared to quadratic-planar platinum(ii), allowing for fine-tuning of physicochemical properties such as solubility, lipophilicity, and reduction behavior. These additional ligands can also be used to further enhance drug efficacy by attachment of targeting moieties or additional bioactive ligands.^8^

In modern cancer chemotherapy, platinum(ii) compounds are rarely employed as monotherapy, but are usually administered in combination with other drugs such as taxanes, topoisomerase inhibitors, targeted therapies or antimetabolites.^9^ One example of the last category is gemcitabine (2′,2′-difluoro-2′deoxycytidine), an antimetabolite widely used in the treatment of different cancer types.^10^ As gemcitabine is already approved in combination with cis- or carboplatin for bladder, non-small cell lung, and ovarian cancer,^11,12^ it is an ideal candidate for direct axial attachment to platinum(iv) prodrugs. Recently, Gibson *et al.* investigated the first gemcitabine-cisplatin(iv) complexes (with phenylbutyrate as second bioactive axial ligand), revealing slightly increased effectiveness compared to the combined administration of the free drugs in the Lewis lung carcinoma model, while at the same time showing less toxicity.^13^

However, a general problem of many platinum(iv) complexes is their insufficient tumor-targeting and accumulation properties. Macromolecular passive targeting strategies offer the advantage of longer plasma half-life time while simultaneously exploiting the enhanced permeability and retention (EPR) effect.^14,15^ In more detail, due to the fast growth and altered signaling processes of the solid tumor tissue, blood vessels are often not fully functional and leaky, allowing macromolecules to enter the interstitium. Furthermore, the lymphatic drainage system is faulty, resulting in retention of the macromolecules in the tumor.^16^ An elegant strategy to exploit these specific characteristics of the malignant tumor is the use of natural nanocarriers like human serum albumin.^17^ Albumin is the main blood plasma protein with an exceptionally long half-life of about 19 days. Albumin is actively taken up by cells and often even actively degraded in the cancerous tissue. Noteworthy, albumin has a free cysteine at position 34, which can be utilized as an endogenous binding site for bioactive compounds.^18^ Maleimides are the most frequently used moieties able to covalently and rapidly bind onto this free thiol.^19^ Consequently, during the last years, we focused on the development of maleimide-bearing, albumin-targeting platinum(iv) complexes, which are characterized by improved pharmacological properties, highly increased tumor accumulation and strong anticancer efficacy.^20–22^ As only one of the two axial ligands is needed for albumin-binding, the second one can be used for the design of targeted dual-action platinum(iv) prodrugs, via attachment of an additional synergistic bioactive ligand.^23–25^ Our previous data already indicated that *e.g.* the exact linker type for the attachment of the bioactive ligand/maleimide moiety can distinctly impact the activity of the prodrugs.^26^ In this study, we developed the first maleimide-bearing gemcitabine-platinum(iv) complexes and step-by-step improved their properties based on bioanalytical data and the *in vivo* antitumor activity in mice to generate an optimized prodrug for further preclinical development.

## Results and discussion

**Fig. 1:**
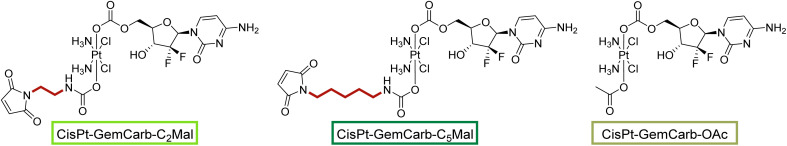
First panel of cisplatin-releasing maleimide-platinum(iv)-gemcitabine complexes with two different maleimide linker lengths.

We started the project with the synthesis of two cisplatin(iv) complexes: CisPt-GemCarb-C_2_Mal and CisPt-GemCarb-C_5_Mal. These two drugs differ in linker length of the maleimide moiety and thus allowed us to analyze the influence of this parameter on hydrolytic stability and albumin-binding rate (Fig. 1). The cisplatin(iv) starting precursor, CisPt(IV)-diBocGemCarb-OH, was synthesized following literature procedures (see Experimental part).^13^ Maleimides were subsequently linked to platinum utilizing isocyanates generated *via* ethyl chloroformate and NaN_3_, or diphenylphosphoryl azide from the respective carboxylic acids. Finally, the boc-protecting groups were removed with trifluoroacetic acid (TFA) and the complexes were purified with preparative high performance liquid chromatography (HPLC) yielding the TFA salts. As maleimides tend to hydrolyze at physiological pH conditions,^27^ they are not suitable *e.g.* for reduction or cell culture studies. Therefore, additionally the acetato-complex CisPt-GemCarb-OAc was synthesized as reference, using acetic anhydride (Fig. 1). All compounds were characterized *via*^1^H- and ^13^C-NMR, mass spectrometry and elemental analysis (see experimental part). All biologically investigated complexes showed high solubility (>5 mM) in aqueous solution.

**Fig. 2:**
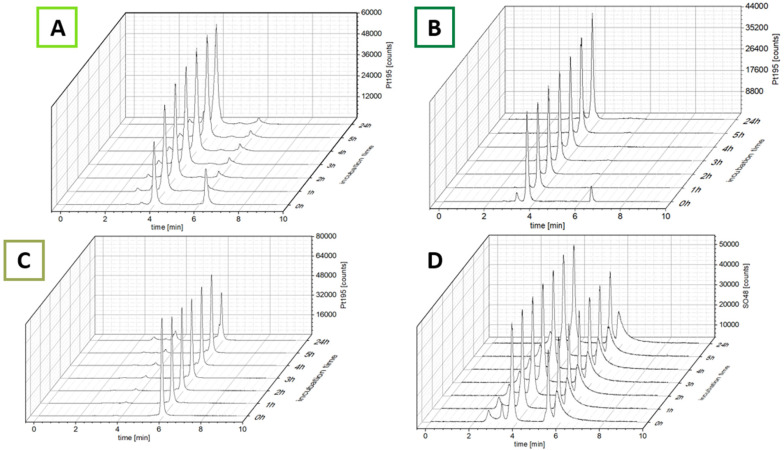
^195^Platinum-traces of CisPt-GemCarb-C_2_Mal (A), CisPt-GemCarb-C_5_Mal (B) and CisPt-GemCarb-OAc (C), incubated in FCS (containing 150 mM phosphate buffer, pH 7.4) at 37 °C, measured with SEC-ICP-MS. (D) ^48^Sulfur trace of experiment (A).

The ability of the complexes to bind to albumin was investigated using size-exclusion chromatography coupled with inductively coupled plasma mass spectrometry (SEC-ICP-MS). After dissolving the complexes in fetal calf serum (FCS; buffered with 150 mM phosphate buffer to ensure a stable pH of 7.4 over 24 h), the samples were incubated at 37 °C and ^48^SO and ^195^Pt were measured every hour for 5 h and once after 24 h (Fig. 2). The ^48^SO trace of an untreated serum sample and pure albumin proved elution of albumin at 4.0 min and the albumin dimer at ∼3.4 min (Fig. S1†). The first chromatogram after incubation with the maleimide complexes revealed that even after the short time of sample preparation (30 s), for both complexes most of the platinum was already bound to albumin (Fig. 2; ∼85% for CisPt-GemCarb-C_5_Mal and ∼65% for CisPt-GemCarb-C_2_Mal). After 1 h the initial peaks of unbound complex in the low-molecular weight fraction (LMWF) disappeared. However, for CisPt-GemCarb-C_2_Mal a small peak (∼6%) at slightly lower retention time developed. This can most probably be explained by hydrolysis of the maleimide, which disables albumin binding. For CisPt-GemCarb-C_5_Mal, this new peak only accounted for 2% platinum. Both complexes exhibited stable binding to albumin over 24 h. The reference complex CisPt-GemCarb-OAc, as expected, did not bind initially to albumin and remained in the LMWF (Fig. 2C). However, within 24 h, ∼30% of the platinum were bound to the protein fraction, most likely in an electrostatic manner.

**Fig. 3:**
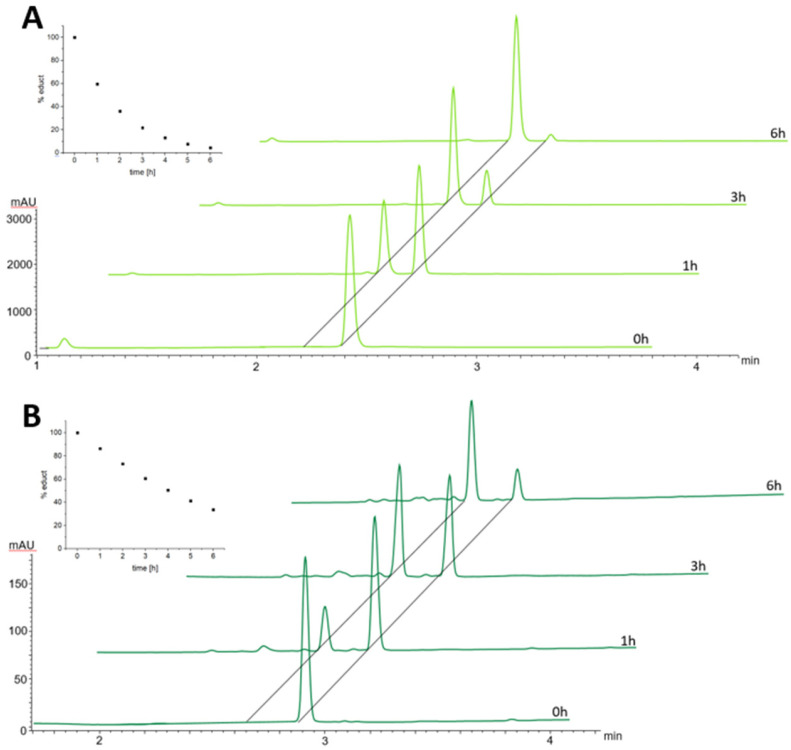
Maleimide hydrolysis of 1 mM CisPt-GemCarb-C_2_Mal (A; 2.4 min) and CisPt-GemCarb-C_5_Mal (B; 2.9 min) in phosphate buffer (150 mM, pH 7.4) at 37 °C, monitored with UHPLC at 220 nm. Inset: decrease of the respective educt in %.

In order to better understand the difference in the albumin-binding rate between the two maleimide complexes, we investigated the hydrolysis behavior of the maleimide moiety through stability studies in phosphate buffer at pH 7.4 and 37 °C (Fig. 3). The maleimide of CisPt-GemCarb-C_2_Mal showed quite fast hydrolysis of about 40% within the first hour (for peak assignment see Fig. S2†). On the other hand, the maleimide of the extended C5 linker of CisPt-GemCarb-C_5_Mal, was only ∼10% hydrolyzed at that time point. After 6 h, the maleimide of CisPt-GemCarb-C_2_Mal was already nearly fully hydrolyzed (∼95%), while ∼30% of CisPt-GemCarb-C_5_Mal remained intact. These results are in line with literature data on the hydrolysis of different maleimide linkers^27^ and can explain the observed difference in albumin-binding rate (Fig. 2). CisPt-GemCarb-OAc, as a non-maleimide reference, revealed no significant changes over 6 h under the same conditions, indicating high stability of the platinum core (Fig. S3A†) and no significant hydrolysis of the carbonate-gemcitabine moiety within this time frame.^28^

**Fig. 4:**
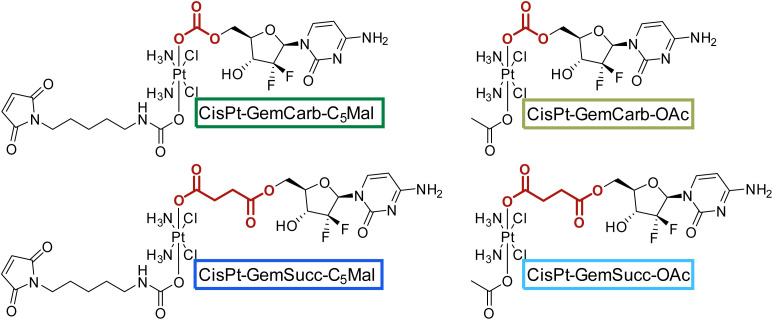
Second panel of maleimide-platinum(iv)-gemcitabine complexes, with two different gemcitabine-platinum linkers (indicated in red).

Knowing that the C5 maleimide linker is preferred, as a next step, the linkage between the platinum core and gemcitabine was modulated. In the first panel (Fig. 1), gemcitabine was directly connected to platinum through a carbonate group. However, previous research demonstrated an alternative approach, where gemcitabine was attached to platinum *via* a succinate linker moiety, resulting in the formation of two ester bonds (CisPt-GemSucc-C_5_Mal; Fig. 4).^13^ For synthesis, in the first step the amine and 3′-OH of gemcitabine were protected again with Boc_2_O. Afterwards, the succinate moiety was coupled *via N*,*N*′-dicyclohexylcarbodiimide and *N*-hydroxysuccinimide.^13^ The formed ligand was coupled to CisPt(iv)(OH)_2_, resulting in CisPt-DiBocGemSucc-OH. Subsequently, the isocyanate of the C5-maleimide was attached, yielding CisPt-DiBocGemSucc-C_5_Mal, which after deprotection and purification, resulted in CisPt-GemSucc-C_5_Mal. Additionally, CisPt-GemSucc-OAc was synthesized as the new acetato reference complex (Fig. 4 and Experimental part).

**Fig. 5:**
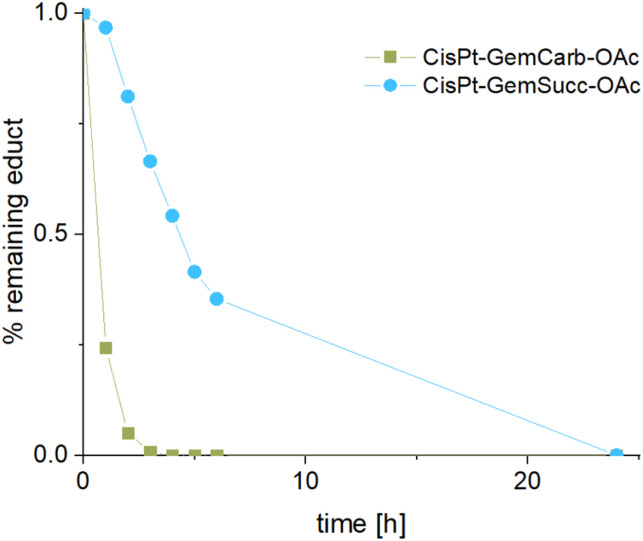
Reduction kinetics of CisPt-GemCarb-OAc and CisPt-GemSucc-OAc at 20 °C with 10 eq. of ascorbic acid over 24 h in phosphate buffer (150 mM, pH 7.4), measured with UHPLC.

The stabilities and maleimide hydrolysis data of the new succinate complexes were comparable to the first panel (Fig. S3A+B and S4†). Next, we were interested, whether the different connectivities influenced the reduction properties. Therefore, CisPt-GemCarb-OAc and CisPt-GemSucc-OAc (1 mM) were dissolved in phosphate buffer (150 mM, pH 7.4) at 20 °C and 10 eq. ascorbic acid were added. The reaction was analyzed with ultra-high performance liquid chromatography (UHPLC) (Fig. 5). The “carbonate” complex CisPt-GemCarb-OAc was completely reduced after only 3 h, while ∼65% of “succinate” analogue CisPt-GemSucc-OAc were still intact at this time point. After 6 h ∼30% CisPt-GemSucc-OAc remained and after 24 h complete reduction was observed. Consequently, in line with literature,^13^ the “carbonate” type linked complex is reduced faster compared to the “succinate” type complex. Furthermore, also lower stability of the “carbonate” type complexes in cell culture medium was reported.^28^ The released gemcitabine and gemcitabine-succinate could also be detected (Fig. S5†). The albumin-binding ability of the maleimide complex CisPt-GemSucc-C_5_Mal (Fig. S6†) was similar to the “carbonate” analogue CisPt-GemCarb-C_5_Mal (Fig. 2).

As a next step, we investigated the biological activities of the new drugs. Cell culture experiments with maleimide-bearing complexes are not feasible due to chemical reactions of the maleimide with the artificially high content of free amino acids in the culture medium. Therefore, we used the maleimide-free platinum(iv) analogs CisPt-GemCarb-OAc and CisPt-GemSucc-OAc (Fig. 4) in comparison to CisPt(OAc)_2_ and cisplatin. As test models, we chose the human HCT116 and murine CT26 colon cancer as well as the intrinsically gemcitabine-resistant human pancreatic cancer cells Panc-1 (Table 1).^29^ Gemcitabine was very active in the nM range (with the exception of Panc-1), while cisplatin had IC_50_ values in the low μM range. The prodrug CisPt(OAc)_2_ was ∼2–10-fold less active than cisplatin. In the colon cancer cell models, the two gemcitabine-releasing platinum complexes behaved more like gemcitabine with IC_50_ values also in the nM range. However, a 2–5-fold reduction in activity compared to gemcitabine was observed. Noteworthy, our new complexes were also able to efficiently circumvent the intrinsic gemcitabine resistance of the Panc-1 cells, resulting in a 3–7-fold increased activity compared to free gemcitabine, with the succinate being more efficient than the carbamate. This indicates that in gemcitabine-sensitive models the platinum core serves as carrier for gemcitabine. In contrast, gemcitabine-resistant models seem to be more affected by the platinum cytotoxicity or are re-sensitized against gemcitabine, resulting in effectivity also in these cancer cell types, thereby highlighting the advantages of multitargeting.

**Table 1:** Anticancer activity, determined by MTT assays after 72 h treatment. IC_50_ values (μM) are given as mean ± SD

Drugs	HCT116	CT26	Panc-1
	IC_50_ (μM) ± SD		
Gemcitabine	0.008 ± 0.002	0.040 ± 0.004	>100
Cisplatin	5.5 ± 1.0	3.4 ± 0.5	10.7 ± 1.6
CisPt(OAc)_2_	68.7 ± 10.3	8.4 ± 0.1	90.9 ± 12.8
CisPt-GemCarb-OAc	0.020 ± 0.002	0.15 ± 0.01	29.7 ± 5.7
CisPt-GemSucc-OAc	0.052 ± 0.002	0.14 ± 0.03	15.7 ± 4.0

**Fig. 6:**
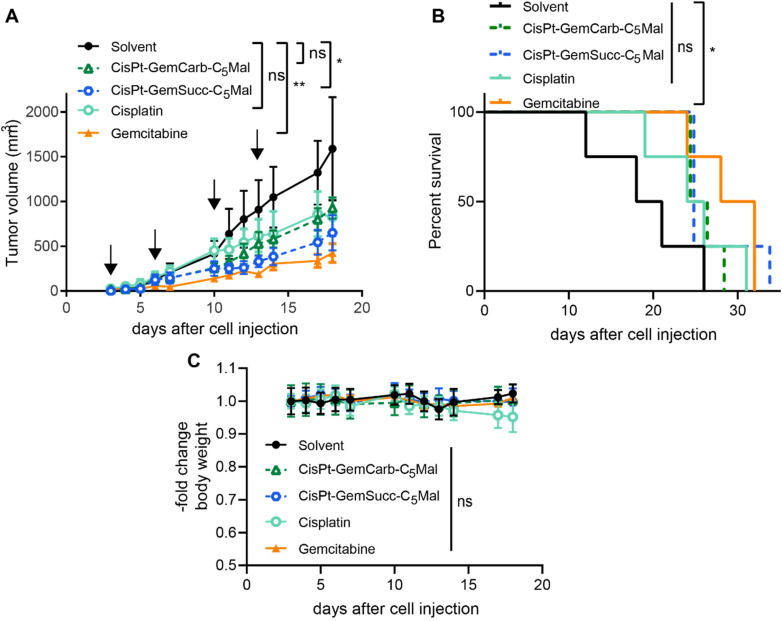
Anticancer activity against CT26 allografts in immune-competent Balb/c mice. (A) Impact on tumor growth. Data are presented as means ± SEM. Arrows indicate i.v. treatments on day 3, 6, 10 and 13. Animals were treated with equimolar concentrations of CisPt-GemCarb-C_5_Mal (10.1 mg kg^−1^ in 0.9% NaCl), CisPt-GemSucc-C_5_Mal (10.5 mg kg^−1^ in 0.9% NaCl), **cisplatin** (3 mg kg^−1^ in 0.9% NaCl) or solvent control (0.9% NaCl). In case of gemcitabine, a dose of 26.2 mg kg^−1^ was used, 10-fold higher compared to the platinum drugs. Significance was calculated using two-way ANOVA and Tukey's multiple comparison test (****p < 0.0001). (B) Overall survival is depicted *via* Kaplan–Meier curve. Significance was calculated using log-rank test and Mantel–Cox posttest (*p < 0.05). (C) Change in body weight of the treated mice.

In order to investigate, whether these effects also translate into the *in vivo* situation, allograft experiments using s.c. CT-26 in immunocompetent Balb/c mice were performed using the respective maleimide-bearing complexes CisPt-GemCarb-C_5_Mal and CisPt-GemSucc-C_5_Mal. The platinum drugs were applied i.v. two-times a week for two weeks at a concentration equimolar to 3 mg kg^−1^ cisplatin (known as the maximal tolerated dose (MTD) for cisplatin). In case of gemcitabine, there is a very strong difference in the activity/toxicity in cell culture (nM range) and *in vivo* with high doses of 60 mg kg^−1^ i.v.^13^ or even 125 mg kg^−1^ i.p.^30^ Consequently, many different therapy schemes are used. Thus, as a compromise, we applied a 10-fold excess of free gemcitabine (26.2 mg kg^−1^) compared to the amount released from the cisplatin(iv) prodrugs. As shown in Fig. 6, CisPt-GemCarb-C_5_Mal displayed anticancer activity similar to cisplatin, which both did not reach statistical significance compared to solvent control. In comparison, CisPt-GemSucc-C_5_Mal showed higher activity (*p* > 0.01 compared to solvent control). These data suggest that the slower reduction of CisPt-GemSucc-C_5_Mal (compare Fig. 5) could correlate with higher *in vivo* anticancer activity. However, the complex was not as active as free gemcitabine alone, which given at a 10-fold excess was the only drug significantly impacting on the overall survival of the CT26-bearing mice. We hypothesized that the released amount of gemcitabine from cisplatin(iv) complexes, when applied at equimolar doses to the MTD of cisplatin, is not sufficient to distinctly improve the *in vivo* anticancer activity. Consequently, we focused our attention on carboplatin(iv) prodrugs because the tolerability of carboplatin is much higher with an MTD of 60 mg kg^−1^ in Balb/c mice and consequently also the applied dose of gemcitabine can be distinctly increased. In addition, a carboplatin core is characterized by extremely slow reduction rates compared to cisplatin,^31^ which could further improve the trend already seen in the cisplatin(iv) panel above.

**Fig. 7:**
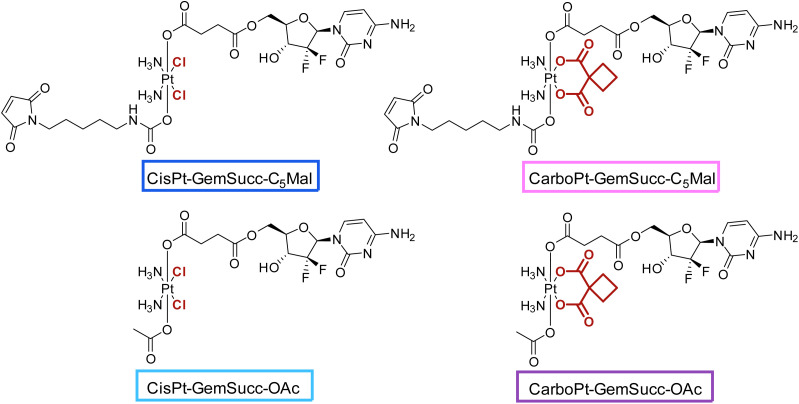
Third panel of maleimide-platinum(iv)-gemcitabine complexes, with two different platinum core structures (cisplatin *vs.* carboplatin).

For the synthesis of the third panel, again DiBocGemSucc-NHS was used and attached to the CarboPt(IV)(OH)_2_ precursor resulting in the asymmetric CarboPt-DiBocGemSucc-OH. Subsequently, similar to the synthetic strategies before (see Experimental part), the isocyanate C_5_-maleimide or the acetate moiety were finally coupled yielding CarboPt-GemSucc-C_5_Mal and its reference compound CarboPt-GemSucc-OAc (Fig. 7).

**Fig. 8:**
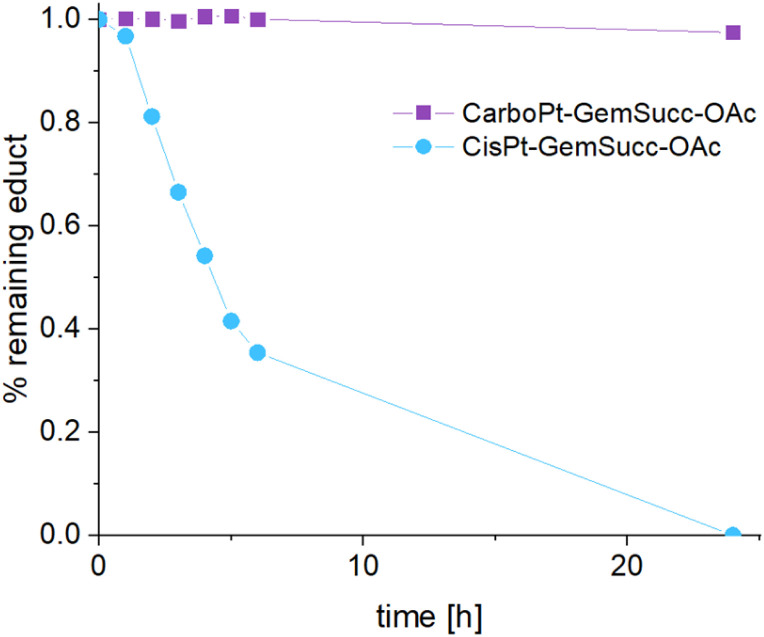
Reduction kinetics of CarboPt-GemSucc-OAc over 24 h in phosphate buffer (150 mM, pH 7.4) at 20 °C with the addition of 10 eq. ascorbic acid, measured with UHPLC (CisPt-GemSucc-OAc data from Fig. 5 was added for comparison).

CarboPt-GemSucc-C_5_Mal also showed fast and stable albumin binding (Fig. S7†), comparable to the cisplatin analogue CisPt-GemCarb-C_5_Mal. Furthermore, the acetato complex CarboPt-GemSucc-OAc showed high stability over >6 h of incubation at 37 °C and pH 7.4 (Fig. S3C†). Regarding reduction kinetics, CarboPt-GemSucc-OAc showed, as desired, massively increased stability with >95% intact complex after 24 h (Fig. 8). At this time point even the more stable CisPt-GemSucc-C_5_Mal was already completely reduced.

Concerning the *in vitro* viability experiments a similar pattern as for the cisplatin complexes was observed with a potent nM activity of CarboPt-GemSucc-OAc in the gemcitabine-sensitive HCT116 and CT26 cells (Table 2). The CarboPt(OAc)_2_ reference complex was basically inactive at the tested concentrations up to 100 μM. Due to the already very low activity of carboplatin, and in accordance with the hypothesis that the activity of the platinum core is central in the mode of action against Panc-1 cells, both carboplatin(iv) complexes were basically inactive in this gemcitabine-resistant model.

**Table 2:** Anticancer activity, determined by MTT assays after 72 h treatment. IC_50_ values (μM) are given as mean ± SD

Drugs	HCT116	CT26	Panc-1
	IC_50_ (μM) ± SD		
Gemcitabine	0.008 ± 0.002	0.040 ± 0.004	>100
Carboplatin	60.9 ± 15.1	20.7 ± 5.9	67.6 ± 0.5
CarboPt(OAc)_2_	>100	87.2 ± 12.6	>100
CarboPt-GemSucc-OAc	0.047 ± 0.007	0.19 ± 0.06	>100

**Fig. 9:**
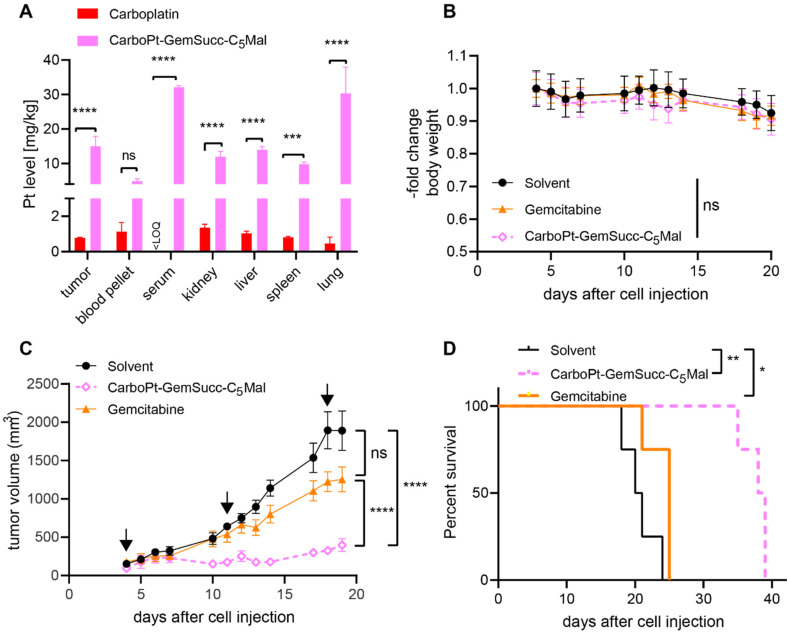
Anticancer activity against CT26 allografts in immune-competent Balb/c mice. (A) ICP-MS measurement of mice treated once i.v. for 24 h with the indicated drug. CarboPt-GemSucc-C_5_Mal (95.9 mg kg^−1^ in 15% propylene glycol (PG) in 0.9% NaCl) was dosed equimolar to 30 mg kg^−1^**carboplatin** (in 0.9% NaCl). Platinum levels in isolated tissues were detected by ICP–MS and normalized to tissue weight. Significance was calculated by two-way ANOVA and Tukey's multiple comparisons test (ns – non significant, ***p < 0.001, ****p < 0.0001). (B) Change in body weight of the treated mice. (C) Impact on tumor growth. Data are presented as means ± SEM. Arrows indicate i.v. treatments (on day 4, 11 and 18) of solvent (15% PG in 0.9% NaCl), CarboPt-GemSucc-C_5_Mal (95.9 mg kg^−1^ in 15% PG in 0.9% NaCl) and **gemcitabine** (21.3 mg kg^−1^ in 0.9% NaCl); all were applied equimolar to 30 mg kg^−1^ carboplatin. Significance was calculated using two-way ANOVA and Tukey's multiple comparison test (****p < 0.0001). (D) Overall survival is depicted *via* Kaplan–Meier curve. Statistical significance was calculated using log-rank test and Mantel–Cox posttest (*p < 0.05, **p < 0.01).

In parallel performed organ distribution data *in vivo* (CarboPt-GemSucc-C_5_Mal was applied at equimolar concentrations to 30 mg kg^−1^ carboplatin) indicated that the maleimide-functionalization resulted not only in a distinctly prolonged plasma half-life but also improved tumor accumulation of the drug 24 h after treatment (Fig. 9A). For a better comparability of the drugs, we again chose the CT26 allograft model for *in vivo* anticancer activity, although previous data indicated that this model is rather carboplatin-resistant (data not shown). Due to the long plasma half-live and high plasma levels of the maleimide drug, we decided on a once a week application scheme. Due to the much higher MTD of carboplatin and to allow a better direct comparability, gemcitabine was applied at equimolar doses and in the same scheme as CarboPt-GemSucc-C_5_Mal. As shown in Fig. 9B, all therapies were well tolerated. CarboPt-GemSucc-C_5_Mal revealed strong and highly significant anticancer activity against the carboplatin-resistant CT26 tumors (Fig. 9C), which resulted in significantly prolonged overall survival of the animals from ∼20 days in the solvent control to ∼40 days (Fig. 9D).

Based on the promising *in vitro* anticancer activity of the cisplatin(iv) drugs against the intrinsically gemcitabine-resistant human Panc-1 cells, we wondered whether the new multi-action complexes were also able to break the acquired gemcitabine-resistance of Capan1/GemR cells.^32^ In cell culture experiments, comparable to data in HCT116 and CT26, the gemcitabine-releasing platinum(iv) acetato complexes had activity in the low nM range against the parental chemo-naïve Capan-1. In contrast, the platinum-only drugs had μM IC_50_ values (Table 3). With regard to the drug resistance, attachment of gemcitabine to the platinum(iv) cores resulted in 3- to 16-fold reduction of the resistance factor of Capan1/GemR as compared to the cytotoxicity of gemcitabine against Capan1 (Table 3).

**Table 3:** Anticancer activity, determined by MTT assays after 72 h treatment. IC_50_ values (μM) are given as mean ± SD

Drugs	Capan1	Capan1/GemR	-fold resistance
	IC_50_ (μM) ± SD		
Gemcitabine	0.029 ± 0.013	83.7 ± 5.1	2790.0
Cisplatin	4.3 ± 0.7	4.2 ± 0.7	1.0
CisPt(OAc)_2_	41.7 ± 13.8	88.8 ± 2.5	2.1
CisPt-GemCarb-OAc	0.12 ± 0.03	20.4 ± 2.3	170.0
CisPt-GemSucc-OAc	0.046 ± 0.011	26.1 ± 8.0	522.0
Carboplatin	50.4 ± 9.7	35.7 ± 2.7	0.7
CarboPt(OAc)_2_	94.7 ± 0.5	>100	>1.0
CarboPt-GemSucc-OAc	0.086 ± 0.002	73.3 ± 17.6	916.3

**Fig. 10:**
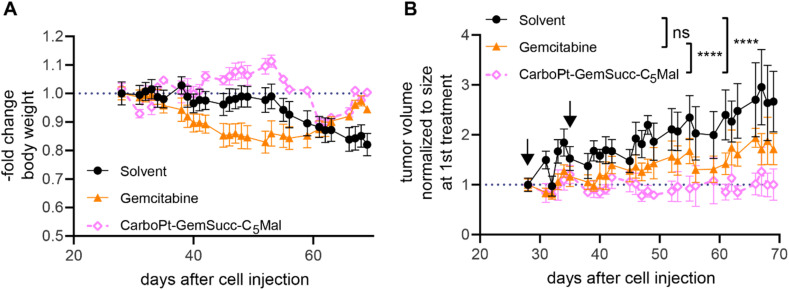
Anticancer activity against Capan1/GemR xenografts in immune-incompetent SCID mice. (A) Change in body weight of the treated mice. (B) Impact on tumor growth. Data are presented as means ± SEM. Arrows indicate i.v. treatments (on day 28 and 35) of solvent (15% PG in 0.9% NaCl), CarboPt-GemSucc-C_5_Mal (95.5 mg kg^−1^ in 15% PG in 0.9% NaCl) or **gemcitabine** (21.3 mg kg^−1^ in 0.9% NaCl) equimolar to 30 mg kg^−1^ carboplatin. Significance was calculated on raw data using two-way ANOVA and Tukey's multiple comparison test (ns – non significant, ****p < 0.0001).

Encouraged by these results, we tested the activity of CarboPt-GemSucc-C_5_Mal in Capan1/GemR xenografts (Fig. 10). Based on the human origin of this cell model, immune-deficient C.B.-17/SCID mice had to be used. In general, Capan1/GemR xenografts are characterized by a very slow tumor growth (only 3-fold increase in tumor volume over 6 weeks) and the occurrence of cachexia (indicated by the continuous loss in body weight of the solvent control, which also finally required the termination of the experiment after 10 weeks). Therapy was started on day 28 (with a scheme comparable to the CT-26 experiment), when the tumors reached a mean size of 100 mm^3^. Capan1/GemR-bearing C.B.-17/SCID mice proofed to be more sensitive to the therapy, indicated by an enhanced loss in body weight upon gemcitabine therapy and even death of one animal in the CarboPt-GemSucc-C_5_Mal group. Consequently, only two cycles of therapy were applied. Noteworthy, in contrast to gemcitabine, all remaining CarboPt-GemSucc-C_5_Mal-treated animals experienced long-lasting stable disease (in some cases even partial remissions) which resulted in a highly significant reduced tumor burden compared to the solvent control. Consequently, beside the limitations of this model, these data indicate that CarboPt-GemSucc-C_5_Mal could be a promising drug to overcome acquired gemcitabine resistance.

## Conclusion

Most clinical anticancer drug treatment schemes contain two or more therapeutics with synergistic activity to optimize the tumor response. However, this can also lead to severe accumulated toxicities, especially in case of chemotherapeutics with low tumor selectivity. Platinum(iv) prodrugs are promising representatives for the development of platinum therapeutics with improved tolerability. Moreover, due to the additional ligands in axial position, other synergistic drugs can be attached. This is especially interesting when using already approved drug combinations like cisplatin/carboplatin with gemcitabine. Noteworthy, the malignant tissue is characterized by an enhanced need for nutrients, thus, increased accumulation of the plasma protein and nutrient carrier albumin is frequently observed. This can be exploited to specifically enrich drugs in the malignant tissue further improving their tumor selectivity. In this study we step-by-step optimized and improved albumin-binding gemcitabine-platinum(iv) complexes based on their bioanalytical behavior and *in vivo* antitumor activity in mice. We started with cisplatin(iv) complexes with gemcitabine as axial ligand and an albumin-binding maleimide moiety. Here a longer maleimide linker (resulting in slower maleimide hydrolysis) and a succinate moiety between the platinum core and gemcitabine (resulting in slower platinum reduction) was preferable. However, due to the frequently higher general toxicity of cisplatin complexes, the amount of gemcitabine possible to be administered *via* the platinum(iv) complex was limited. This problem of drug ratios should in general be considered, especially when developing multi-action cisplatin(iv) drugs. Consequently, a respective gemcitabine-carboplatin(iv) derivative was synthesized, exploiting the distinctly higher tolerability of carboplatin, which also enabled the application of much higher gemcitabine doses. Preliminary *in vivo* experiments in a carboplatin-resistant allograft and a gemcitabine-resistant xenograft revealed significantly improved antitumor activity of this new prodrug. This indicates that the multi-action platinum(iv) approach of combining carboplatin and gemcitabine in one molecule could be a promising strategy for the development of well-tolerable next-generation therapeutics with improved anticancer activity.

## Materials and methods

### Chemicals and instrumentation

**Scheme 1:**
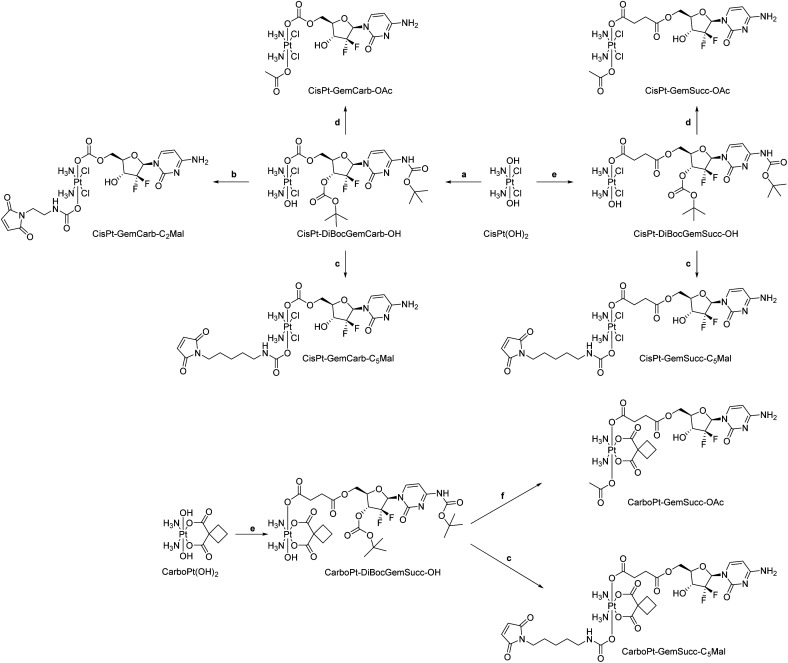
Experimental overview of synthesized complexes. (a) DiBocGem-NHS, DMSO; (b) (1) Mal-C_2_-NCO, DMF (2) CH_2_Cl_2_, TFA; (c) (1) Mal-C_5_-NCO, DMF (2) CH_2_Cl_2_, TFA; (d) (1) Ac_2_O (2) CH_2_Cl_2_, TFA; (e) DiBocGemSucc-NHS, DMSO; (f) (1) Ac_2_O, DMF (2) CH_2_Cl_2_, TFA.

Potassium tetrachloridoplatinate (K_2_[PtCl_4_]) was purchased from Johnson Matthey (Switzerland). Water for synthesis was taken from a reverse osmosis system. For HPLC measurements Milli-Q water (18.2 MΩ cm, Merck Milli-Q Advantage, Darmstadt, Germany) was used. Other chemicals and solvents were purchased from commercial suppliers (Sigma Aldrich, Merck and Fisher Scientific). CisPt-DiBocGemSucc-OH,^13^CisPt-DiBocGemCarb-OH,^13^Mal-C_2_-NCO,^20^Mal-C_5_-NCO,^24^CarboPt(IV)(OH)_2_ ^33^ and DiBocGemSucc-NHS^13^ were synthesized according to literature (Scheme 1).

**Fig. 11:**
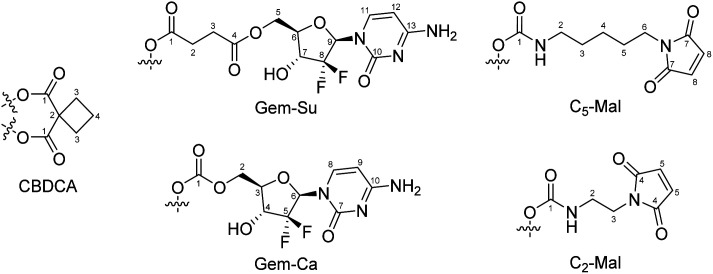
NMR-numbering scheme of ligands.

Analytical HPLC measurements were conducted on a Thermo Scientific Dionex UltiMate 3000 UHPLC-system using a reverse-phase C18 column (Waters Acquity UPLC® BEH C18, 3 × 50 mm, 1.7 μm or Phenomenex Kinetex, 100 × 4.60 mm, 2.6 μm, 100 Å). Milli-Q water, containing 0.1% TFA, and acetonitrile (ACN) containing 0.1% TFA were used as eluents with a gradient of 5–95% over 5 min and a flow rate of 0.6 mL min^−1^ or 1 mL min^−1^, respectively, unless otherwise stated. The compounds were purified by preparative reversed phase (RP)-HPLC using a Phenomenex Luna 250 × 21.2 mm, 10 μm, 100 Å on a Thermo Scientific UltimaMate 3000 system or a Waters XBridge C18 column on an Agilent 1200 Series system. Milli-Q water and acetonitrile were used as eluents with a flow rate of 17 or 15 mL min^−1^ respectively, unless otherwise stated. One- and two-dimensional ^1^H-NMR and ^13^C-NMR spectra were recorded on a Bruker Avance III 500, an AV III 600 or an AV III HD 700 spectrometer at 298 K. For ^1^H- and ^13^C-NMR spectra the solvent residual peak was taken as internal reference. ^195^Pt-NMR were collected on a Bruker AVANCE III HD 500 MHz spectrometer and chemical shifts were reported with respect to K_2_PtCl_4_ in water at −1624 ppm. NMR numbering of the compounds are shown in Fig. 11. Electrospray ionization mass spectra were recorded on a Bruker Amazon SL ion trap mass spectrometer in positive and/or negative mode by direct infusion. High-resolution mass spectra were measured on a Bruker maXis™ UHR electrospray ionization time of flight mass spectrometer. Elemental analysis measurements were performed on a PerkinElmer 2400 CHN Elemental Analyzer at the Microanalytical Laboratory of the University of Vienna. The HPLC chromatograms and NMR spectra of the final complexes are provided in Fig. S8–S30.† In some of the final NMR spectra the typical triplet for ammonium can be observed. This originates from the formation of ammonium trifluoroacetate during preparative HPLC purification, which however did not exceed 2%.

### Synthesis of complexes

#### (OC-6-44)-Acetato-(((2*R*,3*R*,5*R*)-5-(4-amino-2-oxopyrimidin-1(2*H*)-yl)-4,4-difluoro-3-tetrahydrofuran-2-yl)methylcarbonato)diammine-dichloridoplatinum(iv); CisPt-GemCarb-OAc

CisPt-DiBocGemCarb-OH (68 mg, 0.083 mmol) was stirred in acetic anhydride (4 mL). The reaction finished after 3.5 h (monitored by ^195^Pt-NMR; ^195^Pt-shift of the educt: *δ* = 1059.98 ppm) and the solvent was evaporated to dryness under reduced pressure. Methanol was added and the product was precipitated using excess diethyl ether affording CisPt-DiBocGemCarb-OAc with a yield of 52 mg (73%). The solid was re-dissolved in a 1 : 1 mixture of dichloromethane (DCM) and TFA (2 mL) and stirred at room temperature for 15 min (progress of deprotection was monitored using HPLC). A steady stream of air was applied for 20 min to remove the solvents and to afford a sticky yellow solid. Finally, this crude mixture was purified *via* preparative RP-HPLC (Agilent system) with a solvent ratio of H_2_O/ACN (both with 0.1% TFA) = 95/5 and a flow rate of 17 mL min^−1^. The product eluted after about 14 min. The collected fractions were lyophilized. Yield: 24 mg (60%) white powder. ^1^H-NMR (700 MHz, DMSO-*d*_6_): *δ* = 8.69 (bs, 1H, N*H*_2_), 8.10 (bs, 1H, N*H*_2_), 7.73 (d, *J* = 7.74 Hz, 1H, Gem-Ca-8), 6.71–6.39 (b, 7H, N*H*_3_, O*H*), 6.14 (t, *J* = 7.85 Hz, 1H, Gem-Ca-6), 6.02 (d, *J* = 7.96 Hz, 1H, Gem-Ca-9), 4.30 (dd, *J* = 2.15, 12.26 Hz, 1H, Gem-Ca-2), 4.19–4.11 (b, 1H, Gem-Ca-4), 4.14 (dd, *J* = 12.26, 6.02 Hz, 1H, Gem-Ca-2), 4.04 (m, 1H, Gem-Ca-3), 1.93 (s, 3H, C*H*_3_) ppm. ^13^C-NMR (175 MHz, DMSO-*d*_6_): *δ* = 177.95 (*C*OCH_3_), 158.68 (Gem-Ca-1), 158.09 (Gem-Ca-7), 142.62 (Gem-Ca-8), 122.45 (t, *J* = 259.08 Hz, Gem-Ca-5), 95.14 (Gem-Ca-9), 78.12 (Gem-Ca-3), 69.81 (t, *J* = 22.88 Hz, Gem-Ca-4), 65.00 (Gem-Ca-2), 22.39 (CO*C*H_3_) ppm; ^195^Pt-NMR (108 MHz, DMSO-*d*_6_): *δ* = 1231.23 ppm; MS: calcd for [C_12_H_19_Cl_2_F_2_N_5_O_8_Pt − H^+^]^+^ 665.0305, found: 665.0272; elemental analysis calcd for C_12_H_19_Cl_2_F_2_N_5_O_8_Pt·1.5TFA: C: 21.54, H: 2.47, N: 8.37, found: C: 21.22, H: 2.12, N: 8.22.

#### (OC-6-44)-(((2*R*,3*R*,5*R*)-5-(4-amino-2-oxopyrimidin-1(2*H*)-yl)-4,4-difluoro-3-tetrahydrofuran-2-yl)methylcarbonato)diammine-dichlorido(5-(2,5-dioxo-2,5-dihydro-1*H*-pyrrol-1-yl)ethyl)carbamatoplatinum(iv); CisPt-GemCarb-C_2_Mal

Mal-C_2_-NCO (20 mg, 0.121 mmol, 2 eq.) was dissolved in 3 mL dry DMF, CisPt-DiBocGemCarb-OH (50 mg, 0.061 mmol) was added and the mixture was stirred under Ar for 24 h. The solvent was removed under reduced pressure. The crude product was dissolved in 4.5 mL CH_2_Cl_2_, 500 μL TFA were added and the mixture was stirred for 1.5 h. The solvent was removed under reduced pressure. The raw product was dissolved in 6 mL H_2_O (with 0.1% TFA) and purified *via* preparative RP-HPLC (Agilent system) with a solvent ratio of H_2_O/ACN (both with 0.1% TFA) = 90/10 and a flow rate of 17 mL min^−1^. The product eluted after about 10 min. The collected fractions were lyophilized. Yield: 29 mg (60%) white powder. ^1^H-NMR (600 MHz, DMSO-*d*_6_): *δ* = 8.55 (bs, 1H, N*H*_2_), 7.99 (bs, 1H, N*H*_2_), 7.72 (d, *J* = 7.72 Hz, 1H, Gem-Ca-8), 6.99 (s, 2H, C_2_-Mal-5), 6.80 (b, 1H, N*H*), 6.69–6.51 (b, 6H, N*H*_3_), 6.47 (b, 1H, O*H*), 6.14 (t, *J* = 8.28 Hz, 1H, Gem-Ca-6), 5.99 (d, *J* = 7.15 Hz, 1H, Gem-Ca-9), 4.30 (d, *J* = 12.04 Hz, 1H, Gem-Ca-2), 4.19–4.10 (b, 1H, Gem-Ca-4), 4.14 (dd, *J* = 12.34, 6.36 Hz, 1H, Gem-Ca-2), 4.04 (m, 1H, Gem-Ca-3), 3.05 (q, *J* = 6.34 Hz, 2H, C2-Mal-2) ppm. ^13^C-NMR (150 MHz, DMSO-*d*_6_): *δ* = 171.64 (C_2_-Mal-4), 163.93 (Gem-Ca-10), 159.08 (Gem-Ca-1), 142.93 (Gem-Ca-8), 135.01 (C_2_-Mal-5), 95.56 (Gem-Ca-9), 78.55 (Gem-Ca-3), 70.46 (Gem-Ca-4), 65.54 (Gem-Ca-2), 37.83 (C_2_-Mal-2) ppm; MS: calcd for [C_17_H_23_Cl_2_F_2_N_7_O_10_Pt − H^+^]^+^ 789.0572, found: 789.0558; elemental analysis calcd for C_17_H_23_Cl_2_F_2_N_7_O_10_Pt·2H_2_O·TFA: C: 24.29, H: 3.00, N: 10.44, found: C: 24.08, H: 2.74, N: 10.28.

#### (OC-6-44)-(((2*R*,3*R*,5*R*)-5-(4-amino-2-oxopyrimidin-1(2*H*)-yl)-4,4-difluoro-3-tetrahydrofuran-2-yl)methylcarbonato)diammine-dichlorido(5-(2,5-dioxo-2,5-dihydro-1*H*-pyrrol-1-yl)pentyl)carbamatoplatinum(iv); CisPt-GemCarb-C_5_Mal

Mal-C_5_-NCO (32 mg, 0.153 mmol, 2.5 eq.) was dissolved in 3 mL dry DMF, afterwards CisPt-DiBocGemCarb-OH (50 mg, 0.061 mmol) was added and the mixture was stirred under Ar for 24 h. The solvent was removed under reduced pressure. The crude product was dissolved in 3.6 mL CH_2_Cl_2_, 400 μL TFA were added and the mixture was stirred for 2 h at 40 °C. The solvent was removed under reduced pressure. The raw product was dissolved in 250 μL DMSO, 2 mL ACN and 8 mL H_2_O (with 0.1% TFA) and purified *via* preparative RP-HPLC (Agilent system) with a solvent ratio of H_2_O/ACN (both with 0.1% TFA) = 82/18 and a flow rate of 17 mL min^−1^. The product eluted after about 12 min. The collected fractions were lyophilized. Yield: 28 mg (55%) white powder. ^1^H-NMR (700 MHz, DMSO-*d*_6_): *δ* = 7.61 (d, *J* = 7.53 Hz, 1H, Gem-Ca-8), 7.57 (bs, 1H, N*H*_2_), 6.99 (s, 2H, C_5_-Mal-8), 6.63 (b, 6H, N*H*_3_), 6.42 (b, 1H, N*H*), 6.14 (b, 1H, Gem-Ca-6), 5.87 (d, *J* = 7.65 Hz, 1H, Gem-Ca-9), 4.28 (dd, *J* = 12.15, 2.26 Hz, 1H, Gem-Ca-2), 4.18–4.09 (b, 1H, Gem-Ca-4), 4.13 (dd, *J* = 12.26, 6.45 Hz, 1H, Gem-Ca-2), 3.99 (m, 1H, Gem-Ca-3), 2.87 (d, *J* = 6.02 Hz, 2H, C_5_-Mal-6), 2.51 (m, 2H, C_5_-Mal-2), 1.46 (qui, *J* = 7.37 Hz, 2H, C_5_-Mal-5), 1.35 (m, 2H, C_5_-Mal-3), 1.18 (qui, 2H, C_5_-Mal-4) ppm; MS: calcd for [C_20_H_30_Cl_2_F_2_N_7_O_10_Pt − H^+^]^+^ 831.1047, found: 831.1015; elemental analysis calcd for C_20_H_30_Cl_2_F_2_N_7_O_10_Pt·0.5 H_2_O·1.5TFA: C: 27.31, H: 3.14, N: 9.69, found: C: 27.09, H: 3.15, N: 10.01.

#### (OC-6-44)-Acetato(4-(((2*R*,3*R*,5*R*)-5-(4-amino-2-oxopyrimidin-1(2*H*)-yl)-4,4-difluoro-3-tetrahydrofuran-2-yl)methoxy)-4-oxobutanoato)diammine-dichloridoplatinum(iv); CisPt-GemSucc-OAc

CisPt-DiBocGemSucc-OH (50 mg, 0.057 mmol) was stirred in 4 mL acetic anhydride and the reaction progress was monitored *via*^195^Pt-NMR (^195^Pt-shift of the educt: *δ* = 1049.19 ppm). After 3.5 h, completion of the reaction was observed and the solvent was evaporated under reduced pressure. Methanol was added and the product was precipitated using excess diethyl ether. The solid was re-dissolved in 2 mL DCM/TFA (1 : 1) and stirred at room temperature for 15 min (progress of deprotection was monitored *via* HPLC). Afterwards, a steady stream of air was applied for 20 min to remove the solvents and to afford a sticky yellow solid. Finally, the crude mixture was purified *via* preparative HPLC (Thermo system) using a gradient of H_2_O/ACN (both with 0.1% TFA) and lyophilized to obtain the final product. Yield: 23 mg (70%) white powder. ^1^H-NMR (700 MHz, DMSO-*d*_6_): *δ* = 8.72 (b, 1H, N*H*_2_), 8.16 (b, 1H, N*H*_2_), 7.75 (d, *J* = 7.96 Hz, Gem-Su-11), 6.49 (b, 6H, N*H*_3_), 6.15 (t, *J* = 7.74 Hz, 1H, Gem-Su-9), 6.01 (d, *J* = 7.74 Hz, 1H, Gem-Su-12), 4.38 (dd, *J* = 12.48, 2.37 Hz, 1H, Gem-Su-5), 4.30 (dd, *J* = 12.47, 6.44 Hz, 1H, Gem-Su-5), 4.22 (m, 1H, Gem-Su-7), 4.07 (m, 1H, Gem-Su-6), 2.54 (m, 2H, Gem-Su-3), 2.51 (partially under solvent peak, Gem-Su-2), 1.91 (s, 3H, C*H*_3_) ppm. ^13^C-NMR (175 MHz, DMSO-*d*_6_): *δ* = 179.23 (Gem-Su-1), 178.18 (O*C*OCH_3_), 172.11 (Gem-Su-4), 158.12 (Gem-Su-10/13), 157.95 (Gem-Su-10/13), 142.95 (Gem-Su-11), 122.29 (t, *J* = 258.60 Hz, Gem-Su-8), 95.16 (Gem-Su-12), 78.01 (Gem-Su-6), 69.98 (t, *J* = 22.89 Hz, Gem-Su-7), 62.70 (Gem-Su-5), 30.29 (Gem-Su-3), 29.57 (Gem-Su-2), 22.81 (CO*C*H_3_) ppm. ^195^Pt-NMR (108 MHz, DMSO-*d*_6_): *δ* = 1229.12 ppm; MS: calcd for [C_15_H_23_Cl_2_F_2_N_5_O_9_Pt − H^+^]^+^ 722.36, found 722.0; elemental analysis calcd for C_15_H_23_Cl_2_F_2_N_5_O_9_Pt·1.5H_2_O·1.5TFA: C: 23.51, H: 3.01, N: 7.62, found: C: 23.22, H: 2.68, N: 7.77.

#### (OC-6-44)-((((2*R*,3*R*,5*R*)-5-(4-amino-2-oxopyrimidin-1(2*H*)-yl)-4,4-difluoro-3-tetrahydrofuran-2-yl)methoxy)-4-oxobutanoato)diammine-dichlorido(5-(2,5-dioxo-2,5-dihydro-1*H*-pyrrol-1-yl)pentylcarbamato)platinum(iv); CisPt-GemSucc-C_5_Mal

CisPt-DiBocGemSucc-OH (60 mg, 0.068 mmol) and Mal-C_5_-NCO (28 mg, 0.134 mmol, 2 eq.) were dissolved in 3 mL dry DMF and the mixture was stirred overnight under Ar. The solvent was removed under reduced pressure. The residue was taken up in 3.6 mL CH_2_Cl_2_, 400 μL of TFA were added and the mixture was stirred for 2 h. The solvent was removed under vacuum and the crude product was purified *via* preparative HPLC. The raw product was taken up in 4 mL ACN and 16 mL H_2_O and purified *via* preparative RP-HPLC (Agilent system), with a solvent ratio of H_2_O/ACN (both with 0.1% TFA) = 79/21 and a flow rate of 17 mL min^−1^. The product eluted after about 10 min. The collected fractions were lyophilized. Yield: 28 mg (55%) white powder. ^1^H-NMR (700 MHz, DMSO-*d*6): *δ* = 8.51 (bs, 1H, N*H*_2_), 8.04 (bs, 1H, N*H*_2_), 7.71 (d, *J* = 7.7 Hz, 1H, Gem-Su-11), 6.99 (s, 2H, C_5_-Mal-8), 6.57 (b, 6H, N*H*_3_), 6.48 (b, 1H, N*H*), 6.16 (b, 1H, Gem-Su-9), 5.98 (d, *J* = 7.74 Hz, 1H, Gem-Su-12), 4.39 (dd, *J* = 12.26, 2.15 Hz, 1H, Gem-Su-5), 4.30 (dd, *J* = 12.37, 6.35 Hz, 1H, Gem-Su-5), 4.21 (m, 1H, Gem-Su-7), 4.07 (m, 1H, Gem-Su-6), 2.86 (b, 2H, C_5_-Mal-2), 2.54 (m, Gem-Su-3), 2.51 (partially under solvent peak, Gem-Su-2), 1.46 (qui, *J* = 7.42 Hz, 2H, C_5_-Mal-5), 1.34 (m, 2H, C_5_-Mal-3), 1.17 (qui, *J* = 7.63 Hz, 2H, C_5_-Mal-4) ppm. ^13^C-NMR (175 MHz, DMSO-*d*_6_): *δ* = 179.21 (Gem-Su-1), 172.14 (Gem-Su-4), 171.13 (C_5_-Mal-7), 142.68 (Gem-Su-11), 134.46 (C_5_-Mal-8), 122.50 (t, *J* = 259.08 Hz, Gem-Su-8), 95.06 (Gem-Su-12), 84.45 (Gem-Su-9), 77.86 (Gem-Su-6), 69.86 (t, *J* = 23.05 Hz, Gem-Su-7), 62.69 (Gem-Su-5), 40.78 (C_5_-Mal-2), 37.08 (C_5_-Mal-6), 30.24 (Gem-Su-3), 29.59 (Gem-Su-2), 29.27 (C_5_-Mal-3), 27.78 (C_5_-Mal-5), 23.55 (C_5_-Mal-4) ppm; MS: calcd for [C_23_H_33_Cl_2_F_2_N_7_O_11_Pt − H^+^]^+^ 887.1309, found: 887.1274; elemental analysis calcd for C_23_H_33_Cl_2_F_2_N_7_O_11_Pt·1.5TFA: C: 29.5, H: 3.28, N: 9.26, found: C: 29.22, H: 3.44, N: 9.31.

#### (OC-6-44)-Diammine(4-(((2*R*,3*R*,5*R*)-5-(4-((*tert*-butoxycarbonyl)amino)-2-oxopyrimidin-1(2*H*)-yl)-3-((*tert*-butoxycarbonyl)oxy)-4,4-difluorotetrahydrofuran-2-yl)methoxy)-4-oxobutanoato)(cyclobutane-1,1-dicarboxylato)hydroxidoplatinum(iv); CarboPt-DiBocGemSucc-OH

DiBocGemSucc-NHS (200 mg, 0.31 mmol, 1.15 eq.) was added to a stirring suspension of CarboPt(IV)(OH)_2_ (110 mg, 0.27 mmol) in DMSO (15 mL). After a day of stirring at room temperature, the reaction mixture was suspended in excess diethyl ether. The formed precipitate was collected by centrifugation, redissolved in methanol and precipitated once again with excess diethyl ether. Yield: 165 mg (63%) off white solid. ^1^H-NMR (500 MHz, DMSO-*d*_6_): *δ* = 10.6 (br, 1H, N*H*Boc), 7.99 (d, *J* = 7.7 Hz, 1H, Gem-Su-11), 7.10 (d, *J* = 7.7 Hz, 1H, Gem-Su-12), 6.30 (t, *J* = 8.2 Hz, 1H, Gem-Su-9), 5.96 (br, 6H, N*H*_3_), 5.29 (br, 1H, Gem-Su-5), 4.44–4.34 (m, 3H, Gem-Su-5, Gem-Su-6, Gem-Su-7), 2.55–2.45 (m, 8H, CBCDA-3, Gem-Su-2, Gem-Su-3), 1.80 (qui, 2H, CBCDA-4), 1.46 & 1.45 (s, 18H, *t*-Bu_Boc_) ppm; ^195^Pt NMR (108 MHz, DMSO-*d*_6_): *δ* = 1750.1 ppm; MS: calcd for [C_29_H_43_F_2_N_5_O_16_Pt − Na^+^]^+^ 973.2218, found: 973.2209.

#### (OC-6-44)-Acetato-diammine(4-(((2*R*,3*R*,5*R*)-5-(4-((*tert*-butoxycarbonyl)amino)-2-oxopyrimidin-1(2*H*)-yl)-3-((*tert*-butoxycarbonyl)oxy)-4,4-difluorotetrahydrofuran-2-yl)methoxy)-4-oxobutanoato)(cyclobutane-1,1-dicarboxylato)platinum(iv); CarboPt-GemSucc-OAc

To a solution of CarboPt-DiBocGemSucc-OH (165 mg, 0.17 mmol) in 5 mL DMF, acetic anhydride (165 μL, 1.75 mmol, 10 eq.) was added and the mixture was stirred at room temperature for 6 h. After the confirmation of completeness of the reaction *via* HPLC, the solvent was removed under vacuum and the resulting oily residue was dissolved in a minimal volume of ACN and added to pre-cooled diethyl ether (45 mL). The resulting precipitate was collected by centrifugation, taken up in methanol and re-precipitated with diethyl ether to obtain crude acetato complex. The solid was re-dissolved in 2 mL DCM/TFA (1 : 1) and stirred at room temperature for 15 min (progress of deprotection was monitored *via* HPLC). Afterwards, a steady stream of air was applied for 20 min to remove the solvents and to afford a sticky yellow solid. The product was taken up in 4 mL H_2_O and purified *via* preparative RP-HPLC (Agilent system) with a solvent ratio of H_2_O/ACN (both with 0.1% TFA) = 88/12 and a flow rate of 17 mL min^−1^. The product eluted after about 9 min. The collected fractions were lyophilized. Yield: 42 mg (23%) as a white solid. ^1^H NMR (500 MHz, DMSO-*d*_6_): *δ* = 8.67 (br, 1H, N*H*_2_), 8.13 (br, 1H, N*H*_2_), 7.73 (d, *J* = 7.34 Hz, 1H, Gem-Su-11), 6.33 (br, 6H, N*H*_3_), 6.16 (t, *J* = 7.91 Hz, 1H, Gem-Su-9), 5.99 (d, *J* = 7.63 Hz, 1H, Gem-Su-12), 4.38 (dd, *J* = 12.52, 2.35 Hz, 1H, Gem-Su-5), 4.28 (dd, *J* = 12.52, 6.12 Hz, 1H, Gem-Su-5), 4.21 (m, 1H, Gem-Su-7), 4.07 (m, 2H, Gem-Su-6, O*H*), 2.55 (m, 2H, Gem-Su-3), 2.50 (under the solvent peak, 6H, Gem-Su-2, CBDCA-3), 1.90 (s, 3H, C*H*_3_), 1.81 (qui, *J* = 8.00 Hz, 2H, CBDCA-4) ppm. ^13^C-NMR (125 MHz, DMSO-*d*_6_): *δ* = 178.41 (Gem-Su-1), 177.17 (O*C*OCH_3_), 176.42 (CBDCA-1), 176.28 (CBDCA-1), 172.08 (Gem-Su-4), 142.74 (Gem-Su-11), 122.52 (t, *J* = 258.45 Hz, Gem-Su-8), 95.16 (Gem-Su-12), 77.91 (Gem-Su-6), 69.88 (t, *J* = 23.52 Hz, Gem-Su-7), 62.59 (Gem-Su-5), 55.57 (CBDCA-2), 31.20 (CBDCA-3), 31.16 (CBDCA-3), 29.94 (Gem-Su-3), 29.37 (Gem-Su-2), 22.41 (CO*C*H_3_), 15.68 (CBDCA-4) ppm. ^195^Pt-NMR (108 MHz, DMSO-*d*_6_): *δ* = 1940.2 ppm; MS: calcd for [C_21_H_29_F_2_N_5_O_13_Pt − H^+^]^+^: 793.1452, found 793.1438; elemental analysis calcd for C_21_H_29_F_2_N_5_O_13_Pt·H_2_O·1.5 TFA: C: 29.37, H: 3.34, N: 7.13, found C: 29.15, H: 3.29, N: 7.40.

#### (OC-6-34)-Diammine(4-(((2*R*,3*R*,5*R*)-5-(4-((*tert*-butoxycarbonyl)amino)-2-oxopyrimidin-1(2*H*)-yl)-3-((*tert*-butoxycarbonyl)oxy)-4,4-difluorotetrahydrofuran-2-yl)methoxy)-4-oxobutanoato)(cyclobutane-1,1-dicarboxylato)(5-(2,5-dioxo-2,5-dihydro-1*H*-pyrrol-1-yl)pentylcarbamato)platinum(iv); CarboPt-GemSucc-C_5_Mal

CarboPt-DiBocGemSucc-OH (120 mg, 0.126 mmol) and Mal-C_5_-NCO (39 mg, 0.187 mmol, 1.5 eq.) were dissolved in 5 mL dry DMF and the mixture was stirred for 24 h at 30 °C under Ar. The solvent was removed under reduced pressure. The residue was taken up in 3.6 mL CH_2_Cl_2_, 400 μL of TFA were added and the mixture was stirred for 3 h at 30 °C. The solvent was removed under vacuum and the crude product was purified *via* preparative HPLC. The raw product was taken up in 4 mL ACN and 16 mL H_2_O were added and purified *via* preparative RP-HPLC (Agilent system) with a solvent ratio of H_2_O/ACN (both with 0.1% TFA) = 80/20 and a flow rate of 17 mL min^−1^. The product eluted after about 10 min. The collected fractions were lyophilized. Yield: 50 mg (42%) white powder. ^1^H NMR (600 MHz, DMSO-*d*_6_): *δ* = 8.90 (b, 1H, N*H*_2_), 8.31 (b, 1H, N*H*_2_), 7.76 (d, *J* = 7.53 Hz, 1H, Gem-Suc-11), 7.00 (s, 2H, C_5_-Mal-8) 6.51 (b, 1H, N*H*), 6.56–6.36 (b, 6H, N*H*_3_), 6.16 (t, *J* = 8.09 Hz, 1H, Gem-Su-9), 6.03 (d, *J* = 7.90 Hz, 1H, Gem-Su-12), 4.38 (dd, *J* = 12.51, 1.97 Hz, 1H, Gem-Su-5), 4.28 (dd, *J* = 12.33, 5.93 Hz, 1H, Gem-Su-5), 4.21 (m, 1H, Gem-Su-7), 4.08 (m, 1H, Gem-Su-6), 2.86 (d, *J* = 5.27 Hz, 2H, C_5_-Mal-2), 2.55 (m, 2H, Gem-Su-2/3), 2.52–2.45 (partially under the solvent peak, 4H, CBDCA-3), 1.80 (qui, *J* = 7.90 Hz, 2H, CBDCA-4), 1.45 (m, 2H, C_5_-Mal-5), 1.33 (m, 2H, C_5_-Mal-3/Gem-Su-2/3), 1.16 (m, 2H, C_5_-Mal-4) ppm. ^13^C-NMR (150 MHz, DMSO-*d*_6_): *δ* = 178.65 (Gem-Su-1), 176.35 (CBDCA-1), 176.18 (CBDCA-1), 172.08 (Gem-Su-4), 171.12 (C_5_-Mal-7), 142.97 (Gem-Su-11), 134.48 (C_5_-Mal-8), 122.50 (t, *J* = 258.18 Hz, Gem-Su-8), 95.07 (Gem-Su-12), 77.97 (Gem-Su-6), 69.82 (t, *J* = 23.25 Hz, Gem-Su-7), 62.53 (Gem-Su-5), 55.34 (CBDCA-2), 40.74 (C_5_-Mal-2), 37.04 (C_5_-Mal-6), 31.70 (CBDCA-3), 30.67 (CBDCA-3), 29.90 (Gem-Su-2/3), 29.36 (Gem-Su-2/3/C_5_-Mal-3), 29.19 (Gem-Su-2/3/C_5_-Mal-3), 27.73 (C_5_-Mal-5), 23.48 (C_5_-Mal-4), 15.67 (CBDCA-4) ppm; MS: calcd for [C_29_H_39_F_2_N_7_O_15_Pt − H^+^]^+^ 959.22, found: 959.28; elemental analysis calcd for C_29_H_39_F_2_N_7_O_15_Pt·1.5 H_2_O·1.5 TFA: C: 33.23, H: 3.79, N: 8.48, found: C: 33.12, H: 3.72, N: 8.63.

### Stability and reduction experiments

Phosphate buffer (250 mM, pH 7.4) containing 1 mM platinum compound was incubated at 37 °C for stability kinetics and at 20 °C with the addition of 10 eq. l-ascorbic acid for reduction kinetics. The reaction was monitored on a Thermo Scientific Dionex UltiMate 3000 UHPLC-system using a Waters Aquity UPLC BEH C18 1.7 μm 3.0 × 50 mm column. Milli-Q water, containing 0.1% formic acid, and acetonitrile containing 0.1% formic acid were used as eluents. A gradient of 5–95% over 5 min was used. For evaluating the current state of the reaction, the peak area of the parental complex was used unless otherwise stated.

### SEC-ICP-MS studies

Fetal calf serum was purchased from Sigma-Aldrich and buffered with 150 mM phosphate at pH 7.4 in order to guarantee a stable pH. The platinum(iv) complexes (5 mM) were dissolved in 150 mM phosphate buffer (pH 7.4) and diluted 1 : 50 in the buffered serum to obtain a final concentration of 100 μM. The samples were then incubated in the autosampler at 37 °C and analyzed every 1 h for 5 h as well after 24 h. Between each sample a pure water blank was measured. For SEC-ICP-MS measurements an Agilent 1260 Infinity system coupled to an Agilent 7800 ICP-MS equipped with a dynamic reaction cell was used. Oxygen (purity 5.5, Messer Austria GmbH, Gumpoldskirchen, Austria) was used as reaction gas. HPLC parameters are given in Table S1† and ICP-MS operation parameters are given in Table S2.†

### Cell culture

All cell cultures were grown at 37 °C in a humidified atmosphere containing 5% CO_2_. HCT116 cells were cultured in McCoy's 5A Modified Media supplemented with 10% FCS (PAA, Linz, Austria) and 2 mM glutamine. The murine (Balb/c) colon cancer cell model CT26 was cultured in DMEM/F12 (1 : 1) supplemented with 10% FCS. Capan1, Capan1/GemR and PANC1 cells were cultured in RPMI 1640 supplemented with 10% FCS. All cells were purchased from the American Type Culture Collection, Manassas, VA, USA, which the exception of Capan1/GemR, which were kindly provided by Karl Quint and Matthias Ocker from the University of Marburg, Germany.^32^ All cell culture media and reagents were purchased from Sigma-Aldrich Austria. The Capan1/GemR cells were selected with 10 μM gemcitabine. The cells were regularly checked for *Mycoplasma* contamination.

### Anticancer activity *in vitro*

Cells were seeded in 96-well microtiter plates and allowed to adhere overnight. Subsequently, cells were exposed in triplicates to the compounds (pre-dissolved in DMSO, final DMSO concentration <0.5%) in the indicated concentrations. After 72 h cell viability was determined using the 3-(4,5-dimethylthiazol-2-yl)-2,5-diphenyltetrazolium bromide (MTT) assay (EZ4U, Biomedia, Vienna, Austria) according to the manufacturer's recommendation. Absorbance was measured at 450 nm (620 nm as a reference) with a Tecan Reader infinite® M200Pro (Tecan Group Ltd, Switzerland). Data were analyzed using Graph Pad prism (version 8.0.1) to calculate IC_50_ values, as a parameter for cytotoxicity resulting in 50% reduction of cell viability compared to the untreated control cells.

### Animals

Experiments were done according to the regulations of the Ethics Committee for the Care and Use of Laboratory Animals at the Medical University Vienna (proposal number BMBWF_66.009_0157_V_3b_2019), the U.S. Public Health Service Policy on Human Care and Use of Laboratory Animals as well as the United Kingdom Coordinating Committee on Cancer Prevention Research's Guidelines for the Welfare of Animals in Experimental Neoplasia. All animals were kept in a pathogen-free environment with a 12 h light dark-cycle with *ad libitum* access to food and water. Every procedure was performed in a laminar airflow under sterile conditions. Welfare of the animals was monitored daily (*e.g.*, body weight, fatigue, food and fluid consumption). Tumor growth and possible side effects of the treatment were evaluated by daily recording the tumor size by caliper measurement and parameters of the animal's overall health conditions. Tumor volumes (mm^3^) were calculated using following formula: length × width^2^/2.

### Anticancer activity in CT-26-bearing BALB/c mice

CT26 cells (5 × 10^5^ cells in 50 μL serum-free medium) were injected subcutaneously (s.c.) into the right flank of 8–16 weeks old male BALB/c mice (Envigo Laboratories, San Pietro Al Natisone, Italy). When the tumors were palpable (day 3–4), therapy treatment started. Animals were treated i.v. with gemcitabine (21.3 mg kg^−1^ or 26.2 mg kg^−1^ in 0.9% NaCl), cisplatin (3 mg/kg in 0.9% NaCl), CisPt-GemCarb-C_5_Mal (10.1 mg kg^−1^ in 0.9% NaCl), CisPt-GemSucc-C_5_Mal (10.5 mg kg^−1^ in 0.9% NaCl) or CarboPt-GemSucc-C_5_Mal (95.9 mg kg^−1^ in 15% PG in 0.9% NaCl). Samples were first dissolved in PG and afterwards diluted with 0.9% NaCl solution to obtain the desired concentrations with 15% PG. Cisplatin-based drugs were applied in concentrations equimolar to 3 mg kg^−1^ cisplatin. The carboplatin-based drug was applied in concentrations equimolar to 30 mg kg^−1^ carboplatin. The solvent control animals received 0.9% NaCl or 15% PG in 0.9% NaCl. Every day, the animals were monitored for the overall health conditions and tumor size was measured regularly by caliper measurement. In case of overall survival experiments, mice were sacrificed by cervical dislocation in the case of a tumor length >20 mm, tumor ulceration or a decreased body weight of ∼20%.

### Anticancer activity in Capan1/GemR-bearing C.B.17/SCID mice

Capan1/GemR cells (1 × 10^6^ cells in 50 μL serum-free medium) were injected s.c. into the right flank of 12–16 weeks old male C.B.17/SCID mice (Envigo Laboratories, San Pietro Al Natisone, Italy). When the tumors were palpable (day 24), therapy treatment started. Animals were treated i.v. with gemcitabine (21.3 mg kg^−1^ in 0.9% NaCl) or CarboPt-GemSucc-C_5_Mal (95.9 mg kg^−1^ in 15% PG in 0.9% NaCl) in concentrations equimolar to 30 mg kg^−1^ carboplatin. The solvent control received 15% PG in 0.9% NaCl only. Every day, the animals were monitored for the overall health conditions and tumor size was measured regularly by caliper measurement.

### Organ distribution in CT26-bearing BALB/c mice

CT-26 cells (5 × 10^5^ cells in 50 μL serum-free medium) were injected s.c. into the right flank of male BALB/c mice. When tumors reached 10 mm length the animals were treated i.v. with equimolar concentrations of CarboPt-GemSucc-C_5_Mal (95.9 mg kg^−1^ in 15% PG in 0.9% NaCl) or carboplatin (30 mg kg^−1^ in 5% glucose). After 24 h, the animals were sacrificed by cervical dislocation and blood was drawn and incubated for 25 min to allow blood clotting. Additionally, tumors as well as organs were collected. To isolate serum and blood pellets, blood was centrifuged for 10 min at 17 900 g at room temperature. The supernatant characterized as serum was transferred to a new tube and centrifuged again to remove residual red blood cells. All collected samples were stored at −20 °C and further processed for platinum measurements *via* ICP-MS. HNO_3_ (67–69%, supra-pur for trace metal analysis, NORMATOM; Distributor: VWR international, Austria) and conc. H_2_O_2_ supra-pur (30%) were used without further purification. Digestion of tissue (approx. 15–30 mg gravimetrically weighted) was performed with 2 mL of approx. 20% nitric acid and 100 μL H_2_O_2_ using an open vessel graphite digestion system (coated graphite heating plate, coated sample holder-top for 25 mL vials, PFA vials and PFA lids; Labter, ODLAB; Distributor: AHF Analysentechnik AG; Germany). Digested samples were diluted in Milli-Q water (18.2 MΩ cm, Milli-Q Advantage, Darmstadt, Germany). The platinum concentration was determined by ICP-MS analysis. Platinum and rhenium standards were derived from CPI International (Amsterdam, The Netherlands). The total platinum content was determined with a quadrupole-based ICP-MS instrument Agilent 7800 (Agilent Technologies, Tokyo, Japan) equipped with the Agilent SPS 4 autosampler (Agilent Technologies, Tokyo, Japan) and a MicroMist nebulizer at a sample uptake rate of approximately 0.2 mL min^−1^. A radio frequency power of 1550 W was used as well as nickel cones. Argon was used as plasma gas (15 L min^−1^) and as carrier gas (∼1.1 L min^−1^). The dwell time was set to 0.1 s and the measurements were performed in 12 replicates with 100 sweeps. Rhenium served as internal standard for platinum. The Agilent MassHunter software package (Workstation Software, version C.01.04, 2018) was used for data processing.

## Conflicts of interest

There are no conflicts to declare.

## Supplementary Material

QI-011-D3QI02032K-s001
