# Supplementary material for: Stepwise optimization of tumor-targeted dual-action platinum(iv)-gemcitabine prodrugs
Source: Inorg Chem Front. 2023 Dec 6;11(2):534–48. doi: 10.1039/d3qi02032k (PMC10790623; doi:10.1039/d3qi02032k)
Supplement: QI-011-D3QI02032K-s001 [file QI-011-D3QI02032K-s001.pdf]

Electronic Supplementary Material (ESI) for Inorganic Chemistry Frontiers.
This journal is © the Partner Organisations 2023

Stepwise Optimization of Tumor-Targeted Dual-Action Platinum(IV)-Gemcitabine Prodrugs

Supporting information

Alexander Kastner[1,5], Theresa Mendrina[1,2], Tomer Babu[3], Subhendu Karmakar[3], Isabella Poetsch[2], Walter Berger[2,4], Bernhard K. Keppler[1,4], Dan Gibson*[3], Petra Heffeter*[2,4], and Christian R. Kowol*[1,4]

[1] University of Vienna, Faculty of Chemistry, Institute of Inorganic Chemistry, Waehringer Str. 42, 1090 Vienna, Austria.

[2] Center of Cancer Research and Comprehensive Cancer Center, Medical University of Vienna, Borschkegasse 8a, 1090 Vienna, Austria.

[3] Institute for Drug Research, School of Pharmacy, The Hebrew University of Jerusalem, 9112102 Jerusalem, Israel

[4] Research Cluster "Translational Cancer Therapy Research", 1090 Vienna, Austria.

[5] University of Vienna, Vienna Doctoral School in Chemistry (DoSChem), Waehringer Str. 42, 1090 Vienna, Austria.

E-Mail: christian.kowol@univie.ac.at; petra.heffeter@meduniwien.ac.at; dang@ekmd.huji.ac.il

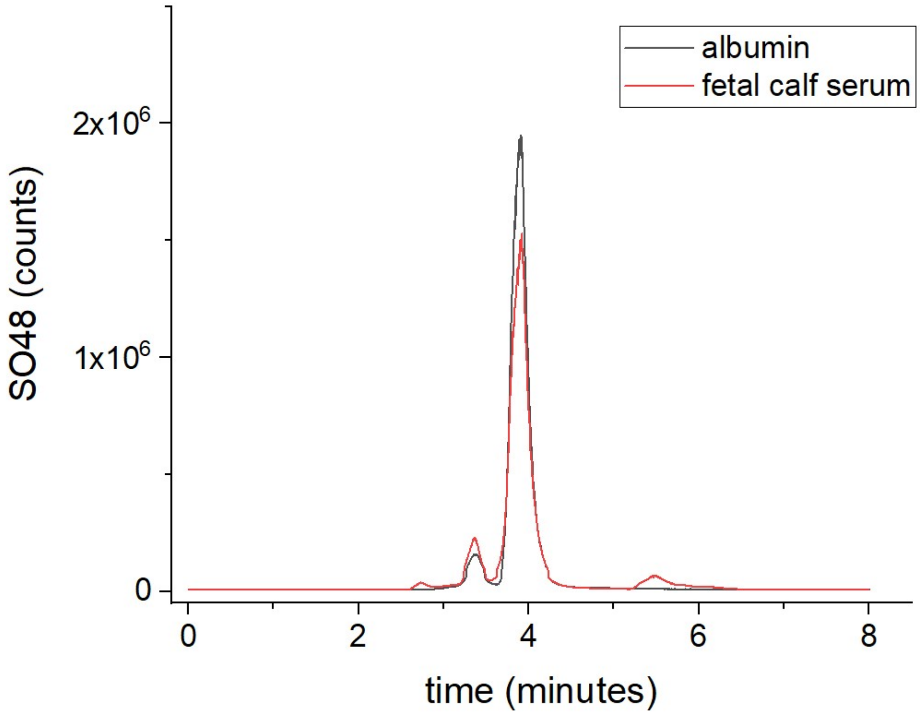

Figure S1: Sulfur traces of fetal calf serum (+150 mM phosphate buffer, pH 7.4) as well as pure albumin in phosphate buffer (50 mM, pH7.4), both measured by SEC-ICP-MS. The small peak at ~3.3 min corresponds to the albumin dimer.

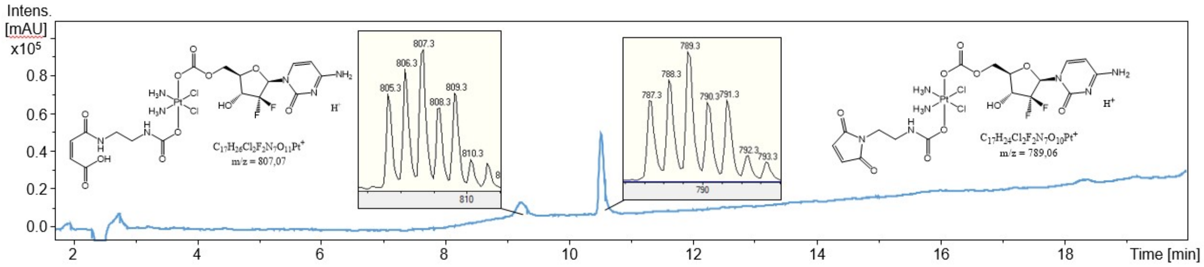

Figure S2: Peak assignment via HPLC-MS of the stability experiment for **CisPt-GemCarb-C$_2$Mal** depicted in Figure 3.

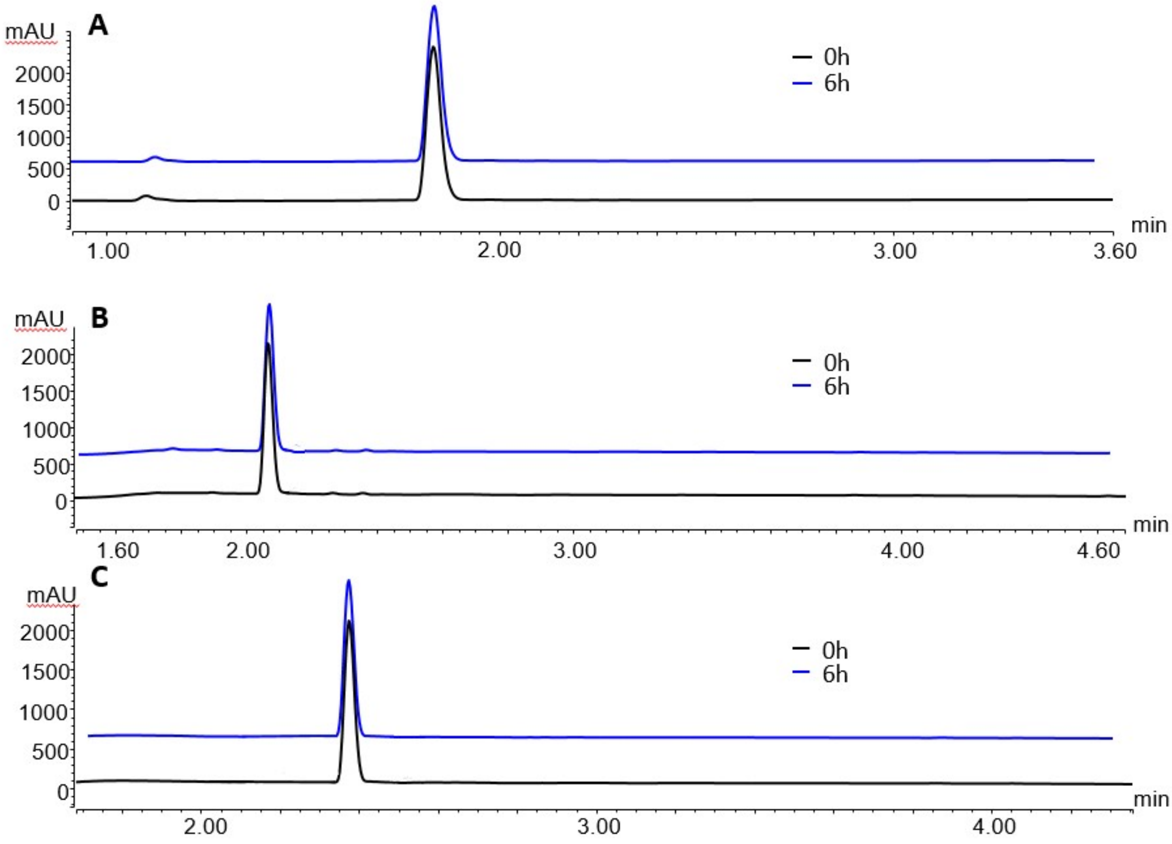

Figure S3. UV/Vis-traces (220 nm) of stability measurements of 1 mM **CisPt-GemCarb-OAc (A), CisPt-GemSucc-OAc (B)** and **CarboPt-GemSucc-OAc (C)** in phosphate buffer (150 mM, pH 7.4) at 37 °C, measured with UHPLC.

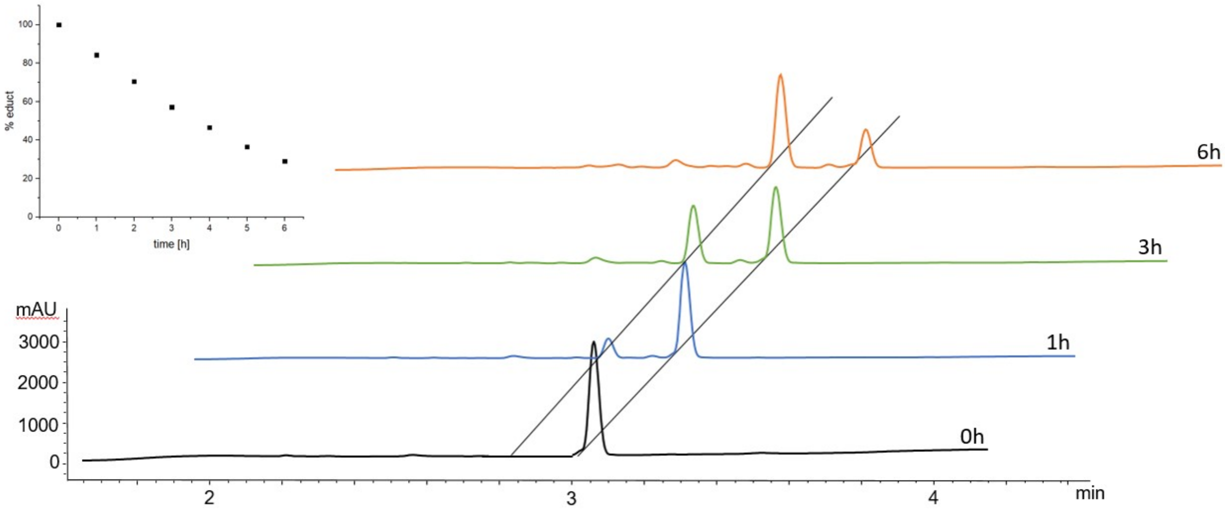

Figure S4. Maleimide hydrolysis of 1 mM **CisPt-GemSucc-C$_5$Mal** in phosphate buffer (150 mM, pH 7.4) at 37 °C, monitored with UHPLC (220 nm).

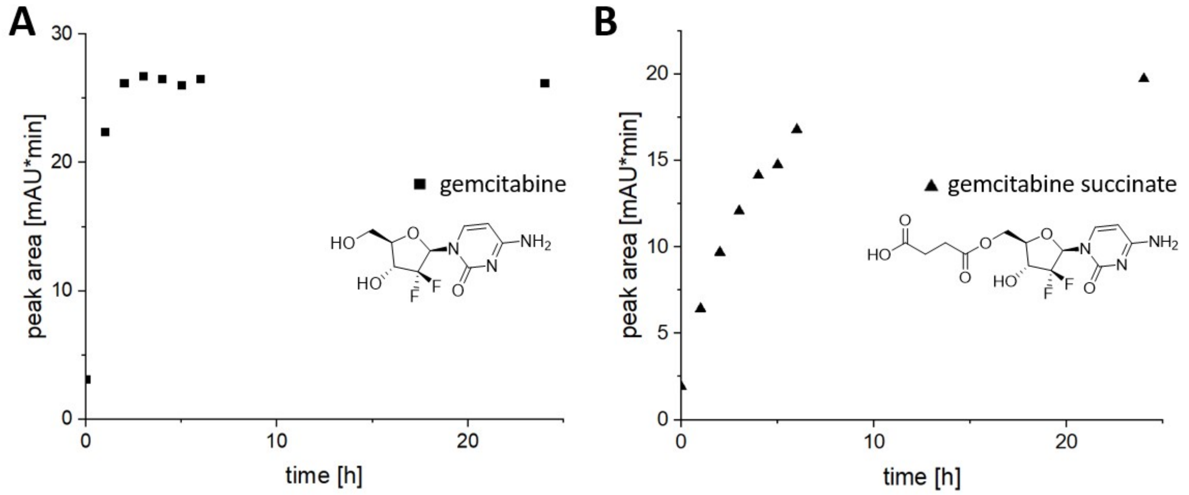

Figure S5: A) Gemcitabine release of the reduction kinetics of **CisPt-GemCarb-OAc** and (B) gemcitabine succinate release of the reduction kinetics of **CisPt-GemSucc-OAc**. The data were measured with UHPLC at 1 mM drug concentration in phosphate buffer (150 mM, pH 7.4) at 20 °C with 10 eq of ascorbic acid over 24 h.

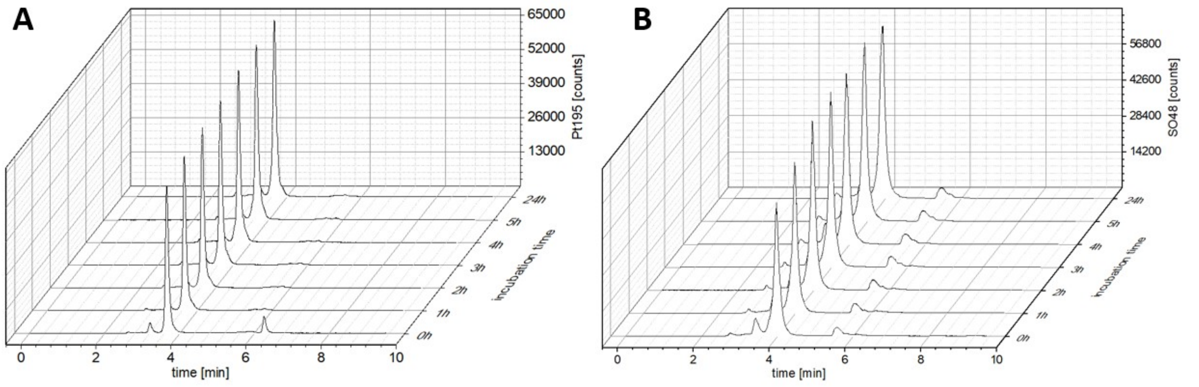

Figure S6: 195Platinum (A) and 48sulfur (B) traces of 100 µM **CisPt-GemSucc-C$_5$Mal** incubated in FCS (buffered with 150 mM phosphate buffer, pH 7.4) at 37 °C, measured with ICP-MS.

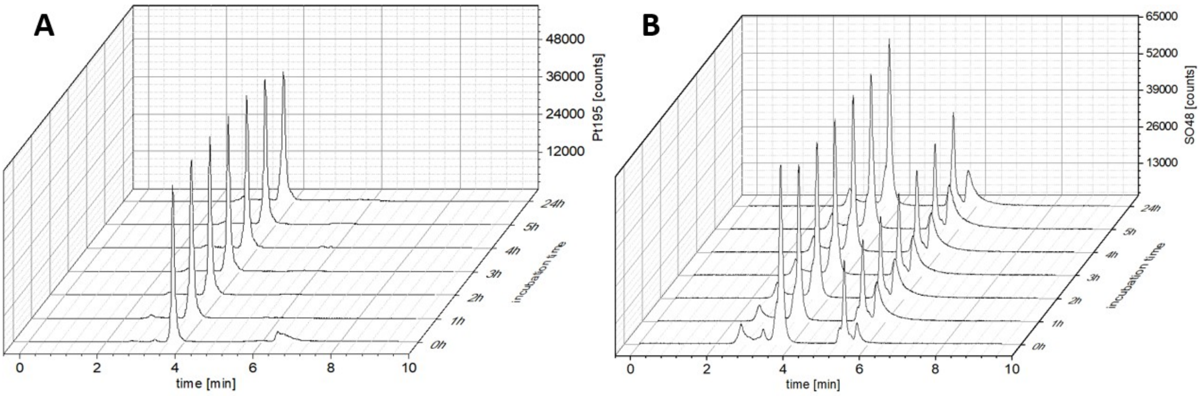

Figure S7: 195Platinum (A) and 48sulfur (B) traces of 100 µM **CarboPt-GemSucc-C$_5$Mal** incubated in FCS (buffered with 150 mM phosphate buffer, pH 7.4) at 37 °C, measured with ICP-MS.

HPLC column:	Acquity UPLC BEH 200Å 1.7 µm, 4.6x150 mm
Eluent:	50 mM CH_3COONH_4, pH = 6.8
Flow rate:	400 µL/min
Injection volume:	0.5 µL
Column temperature:	37 °C
Autosampler temperature:	37 °C

Table S1: SEC-HPLC parameters for SEC-ICP-MS measurements.

Nebulizer:	Quartz
Spray chamber:	Scott type
Nebulizer gas flow:	1.08 L/min
Aux. gas flow:	0.9 L/min
Plasma gas flow:	15 L/min
Reaction gas (oxygen):	30 %
ICP RF power:	1550 W
m/z measured:	195, 48

Table S2: ICP-MS parameters for ICP-MS measurements.

CisPt-GemCarb-OAc

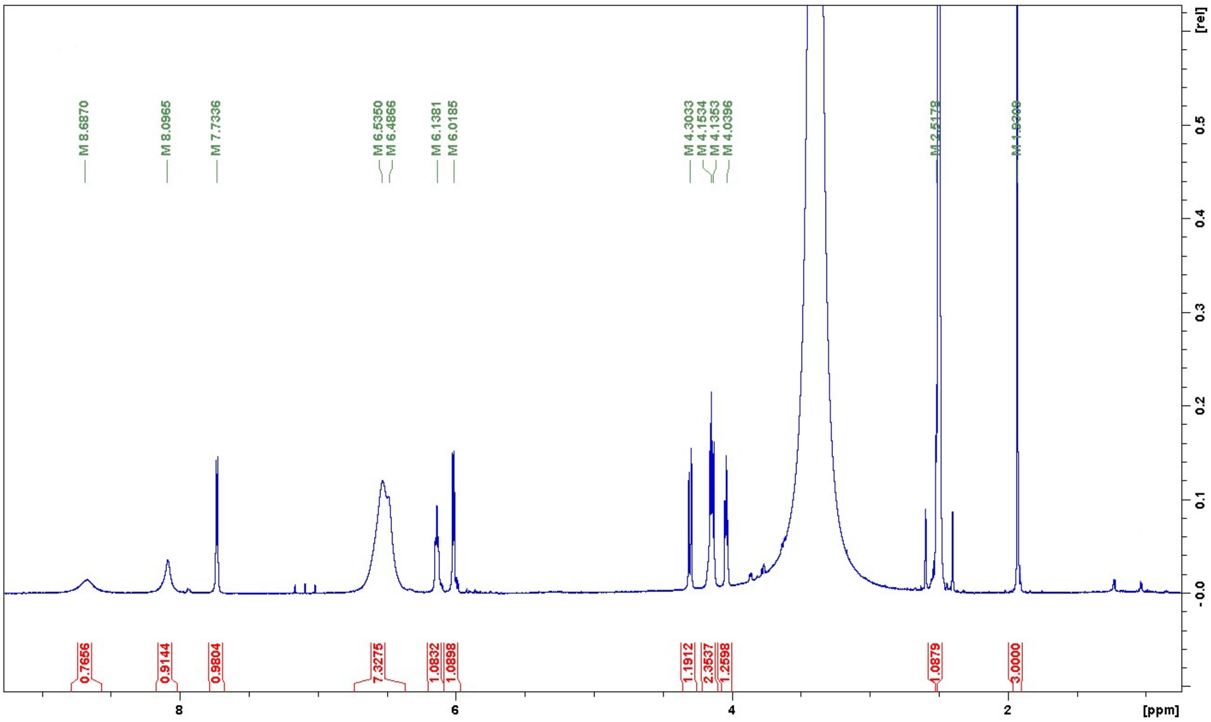

Figure S8: ^{1}H-NMR of CisPt-GemCarb-OAc.

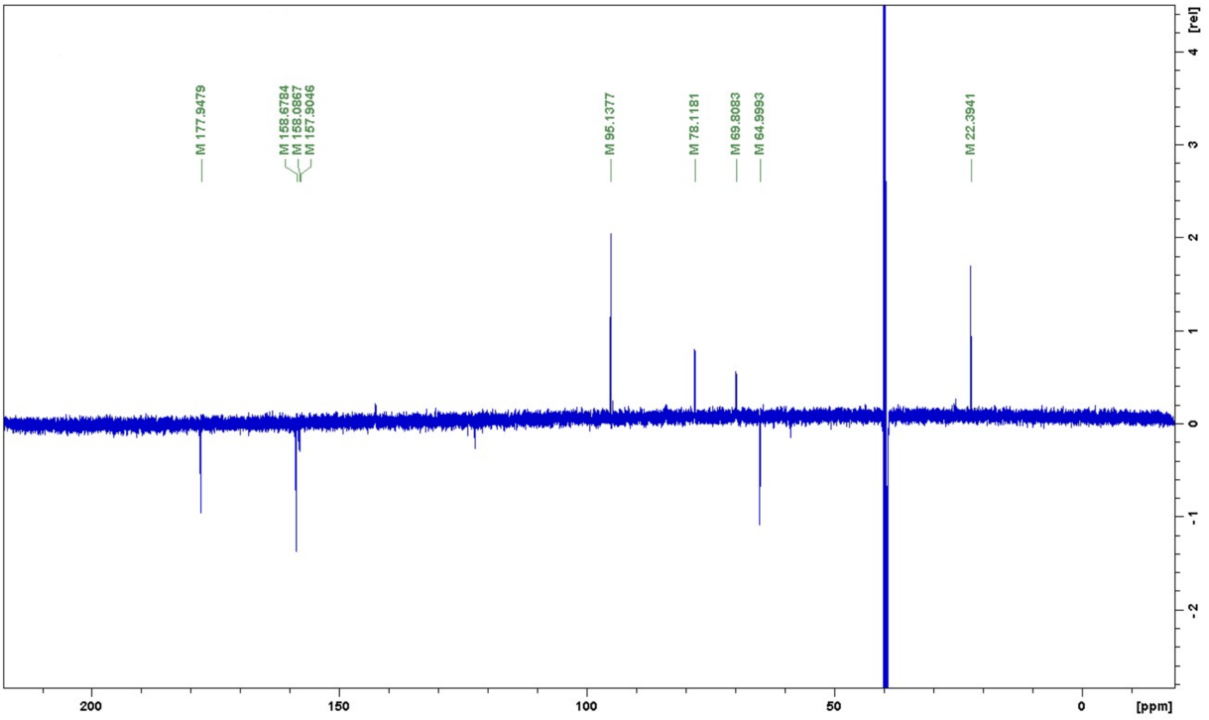

Figure S9: ^{13}C-NMR of CisPt-GemCarb-OAc.

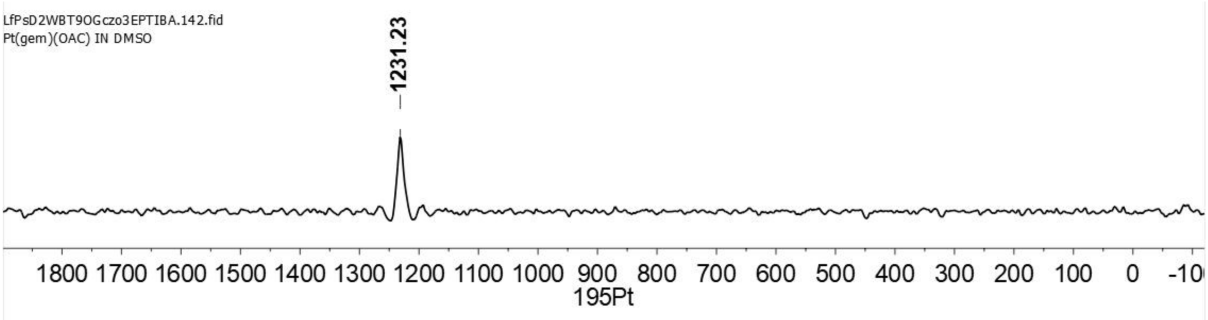

Figure S10: ^{195}Pt-NMR of CisPt-GemCarb-OAc.

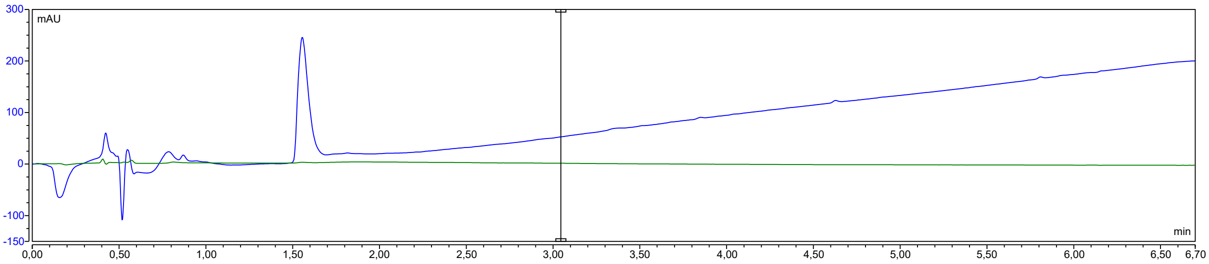

Figure S11: RP-HPLC chromatogram of CisPt-GemCarb-OAc.

CisPt-GemCarb-C_2Mal

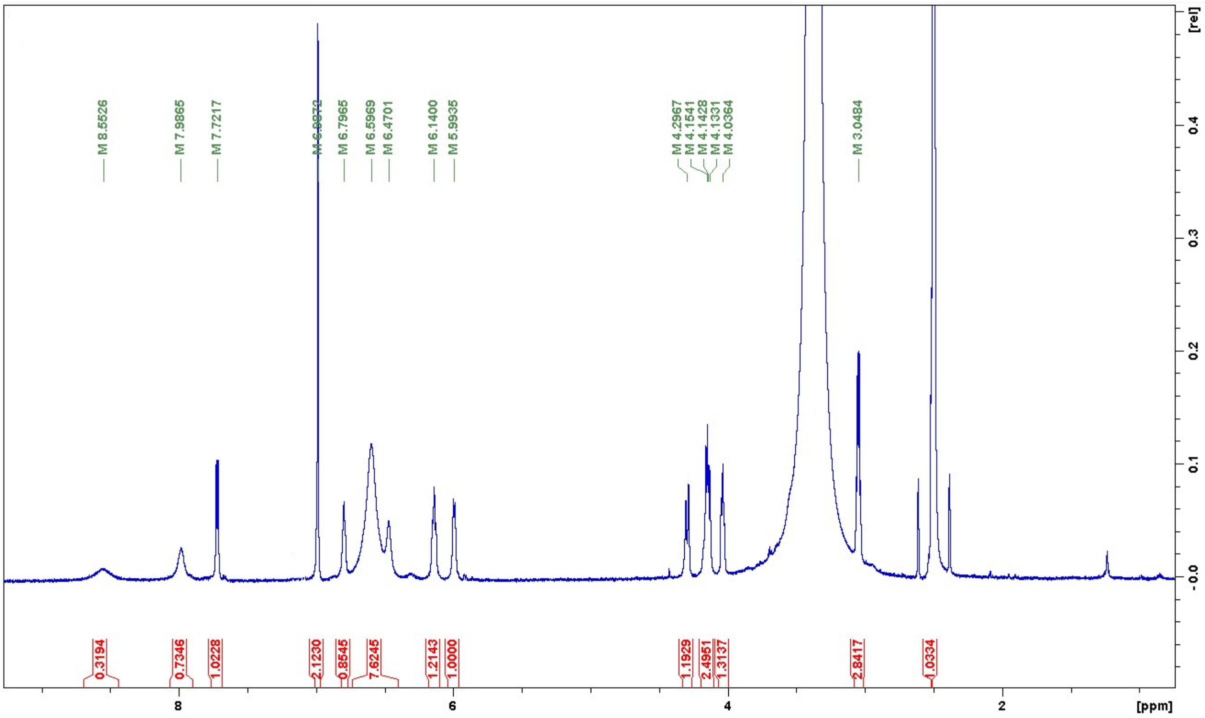

Figure S12: ^{1}H-NMR of CisPt-GemCarb-C_2Mal.

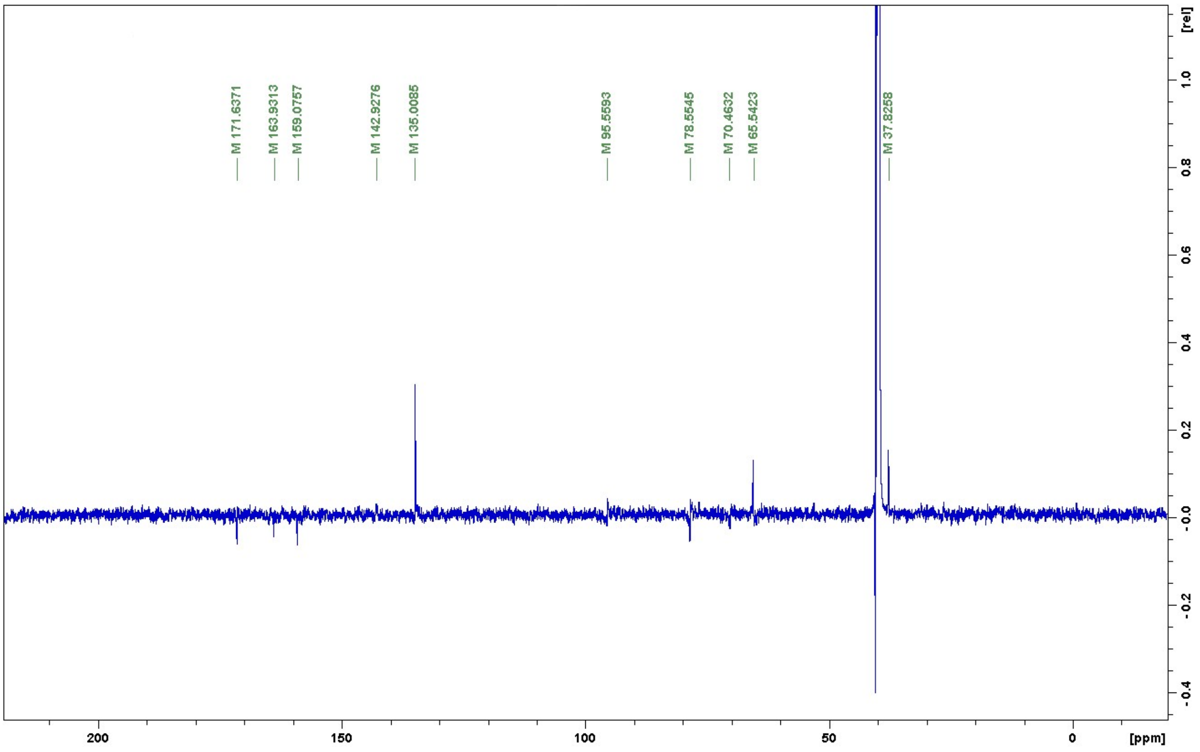

Figure S13: ^{13}C-NMR of CisPt-GemCarb-C_2Mal.

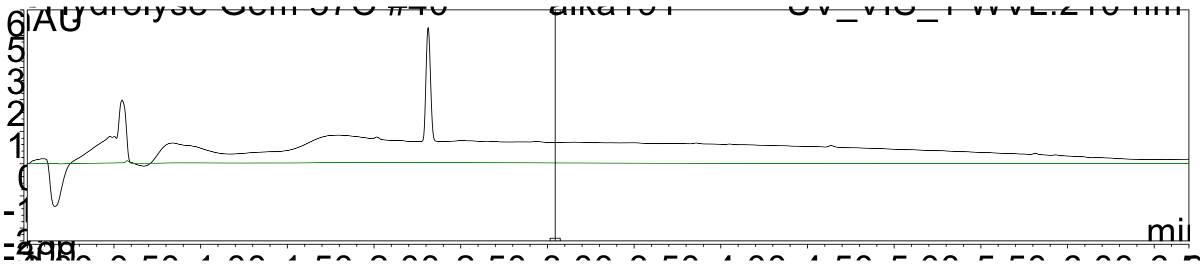

Figure S14: RP-HPLC chromatogram of CisPt-GemCarb-C_2Mal.

CisPt-GemCarb-C_5Mal

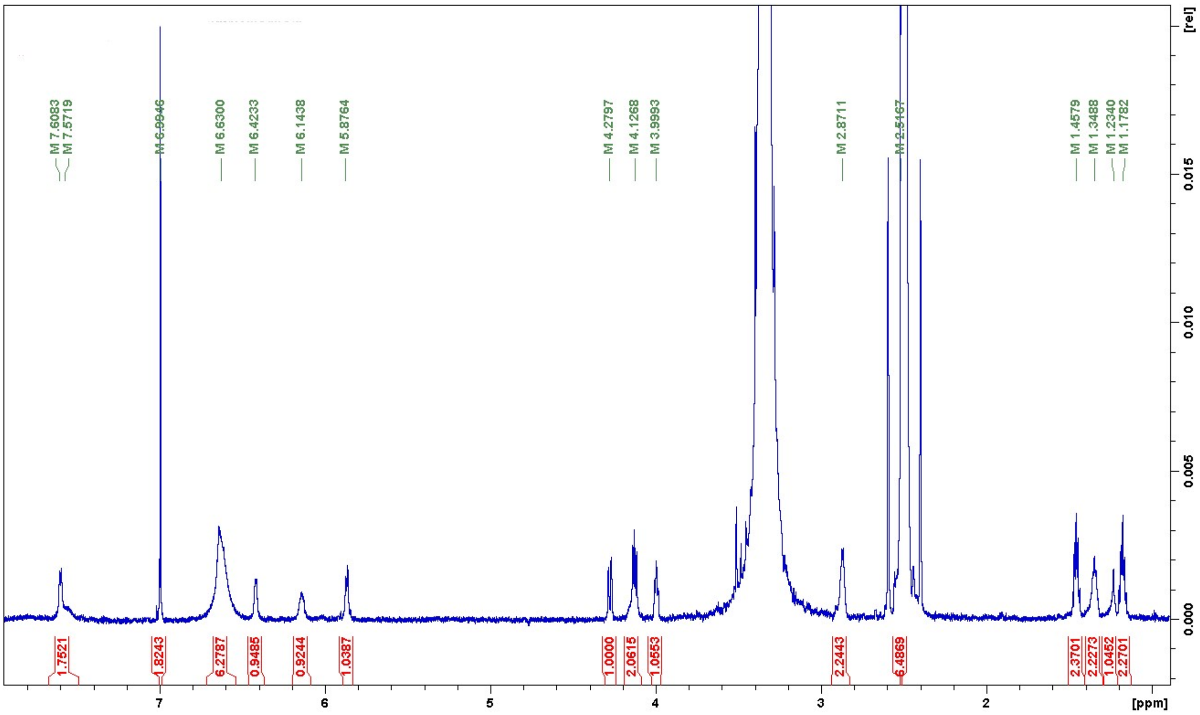

Figure S15: ^{1}H-NMR of CisPt-GemCarb-C_5Mal.

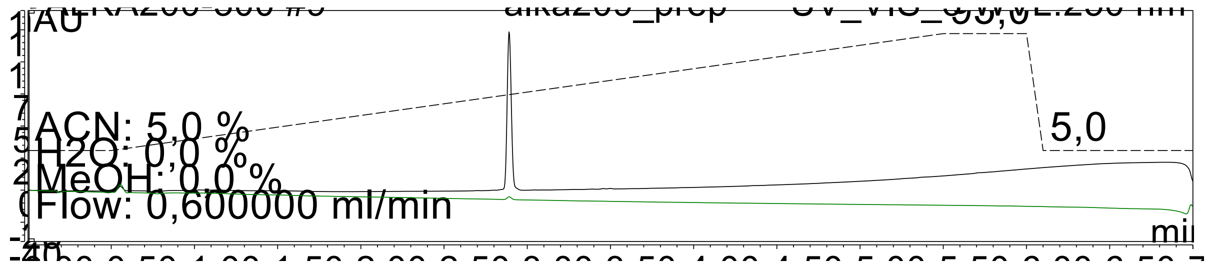

Figure S16: RP-HPLC chromatogram of CisPt-GemCarb-C_5Mal.

CisPt-GemSucc-OAc

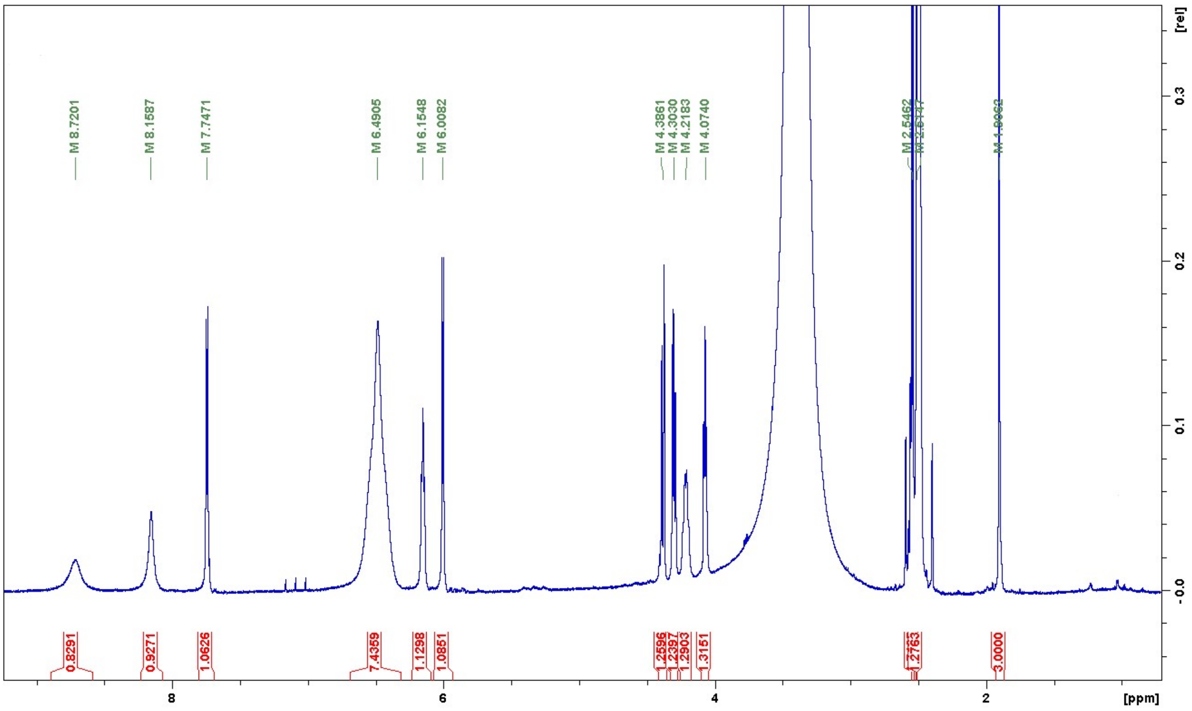

Figure S17: ^{1}H-NMR of CisPt-GemSucc-OAc.

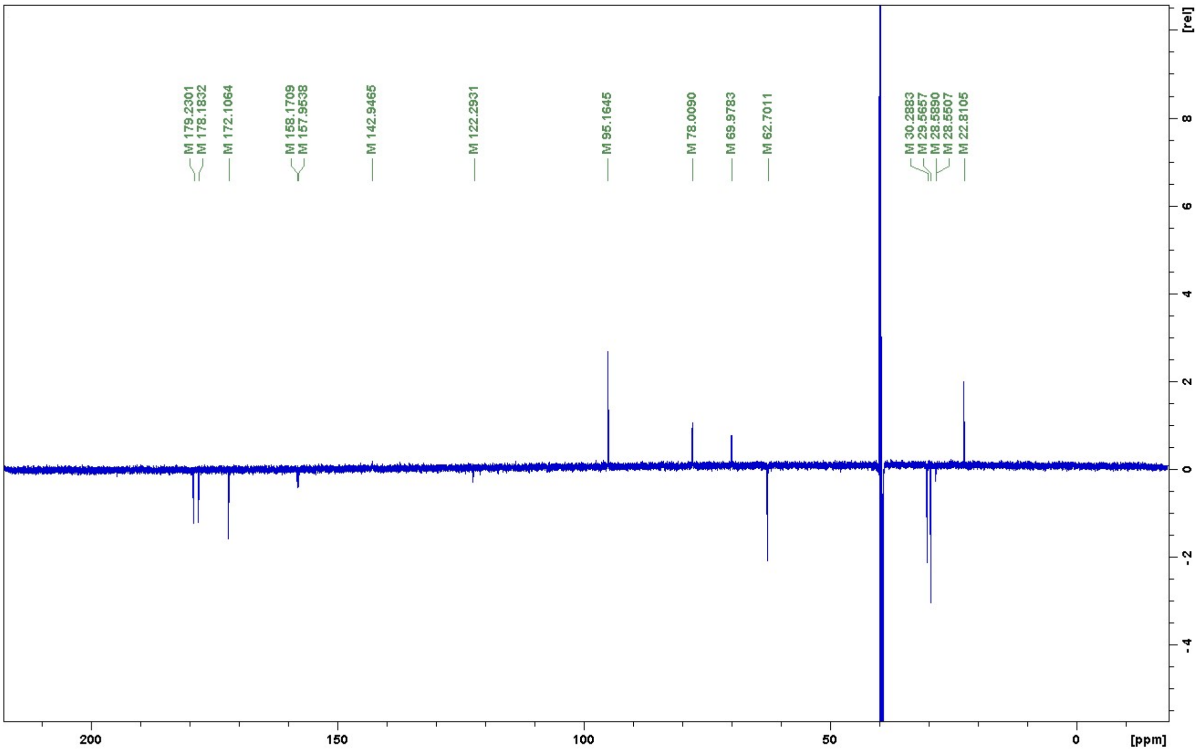

Figure S18: ^{13}C-NMR of CisPt-GemSucc-OAc.

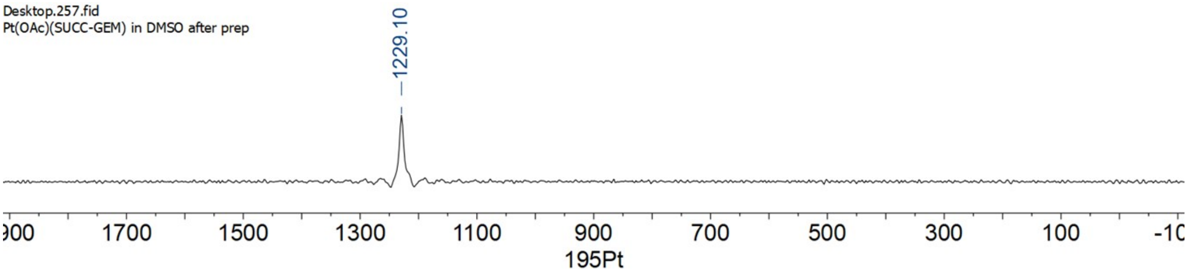

Figure S19: ^{195}Pt-NMR of CisPt-GemSucc-OAc.

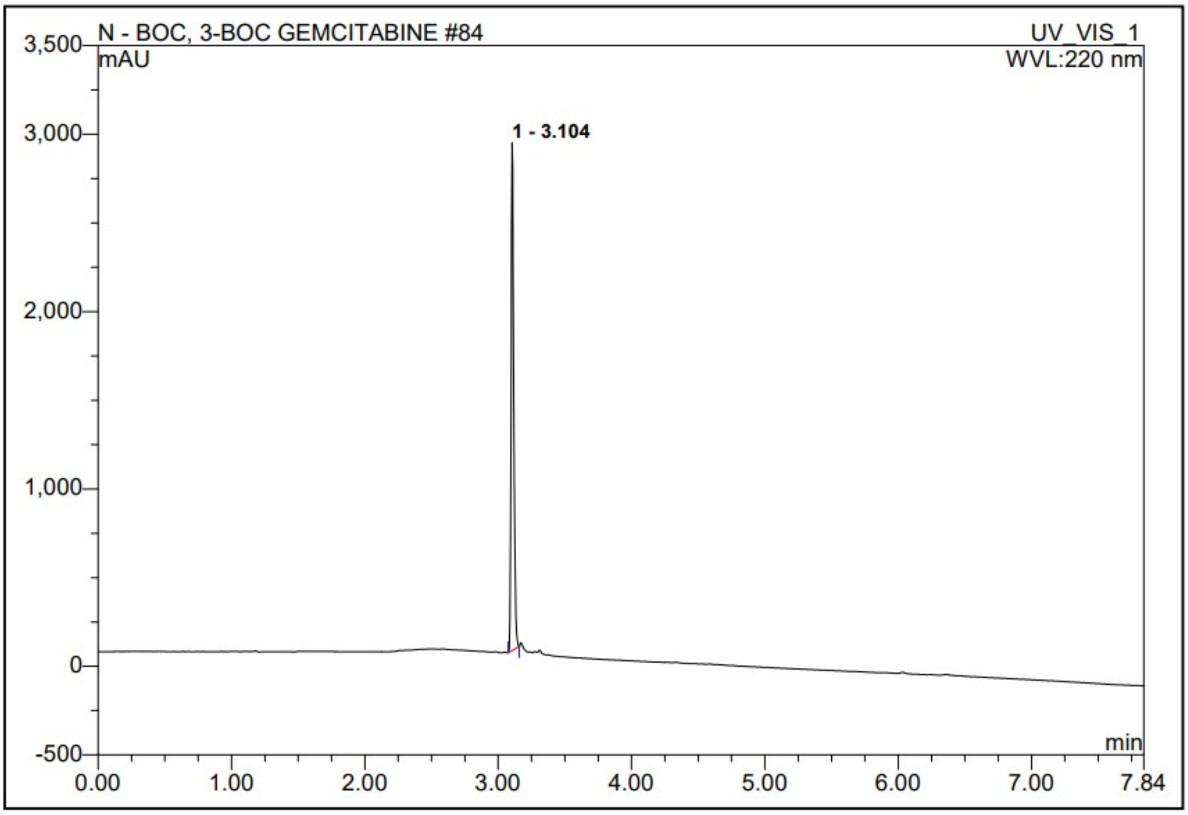

Figure S20: RP-HPLC chromatogram of CisPt-GemSucc-OAc.

CisPt-GemSucc-C$_5$Mal

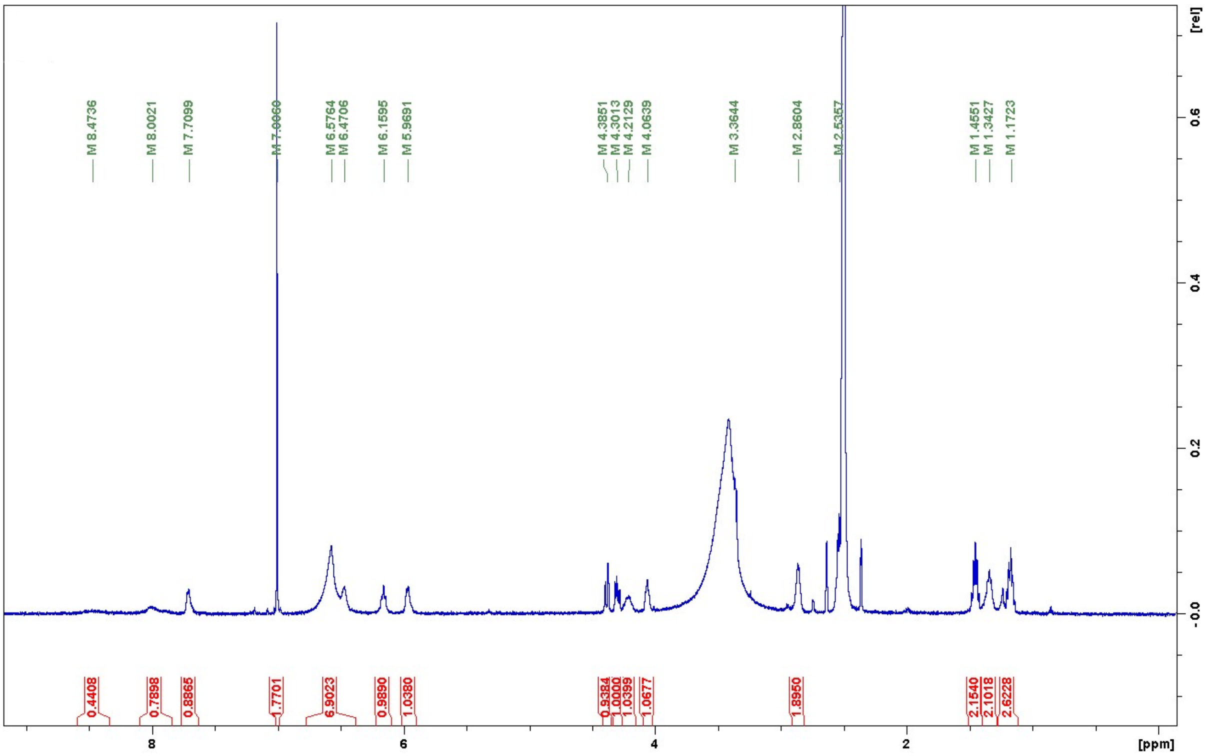

Figure S21: ^{1}H-NMR of CisPt-GemSucc-C$_5$Mal.

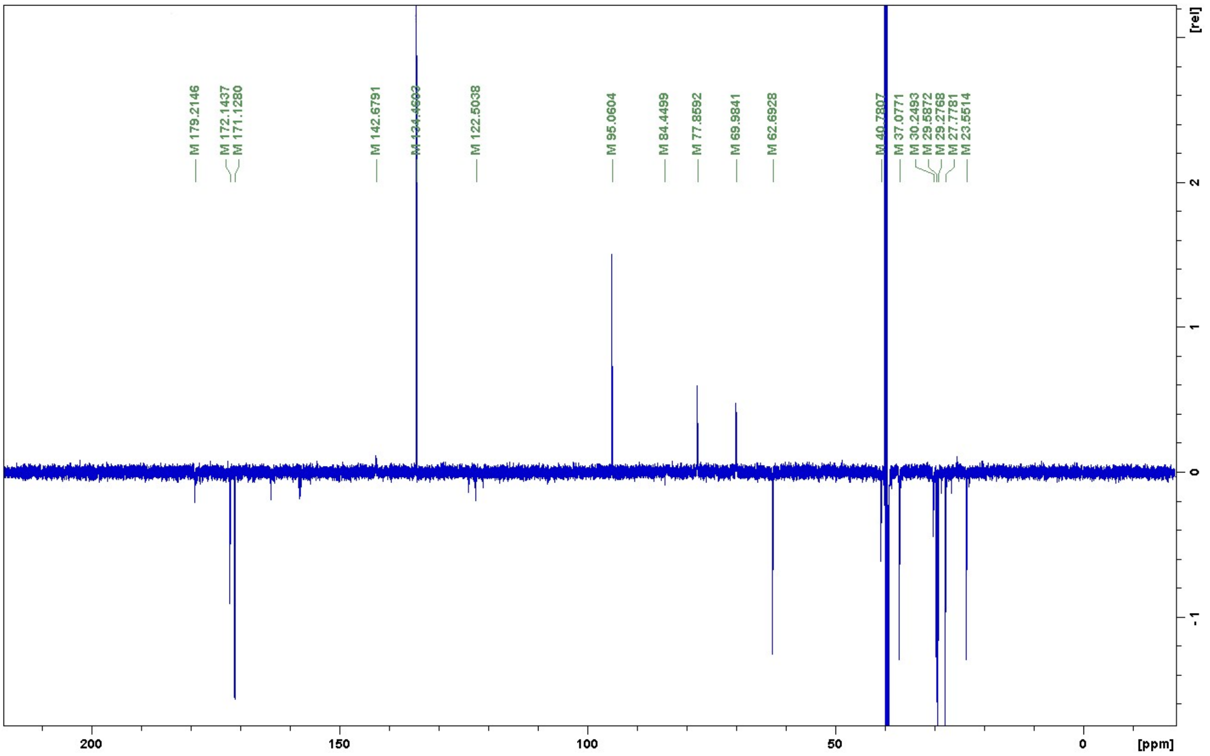

Figure S22: ^{13}C-NMR of CisPt-GemSucc-C$_5$Mal.

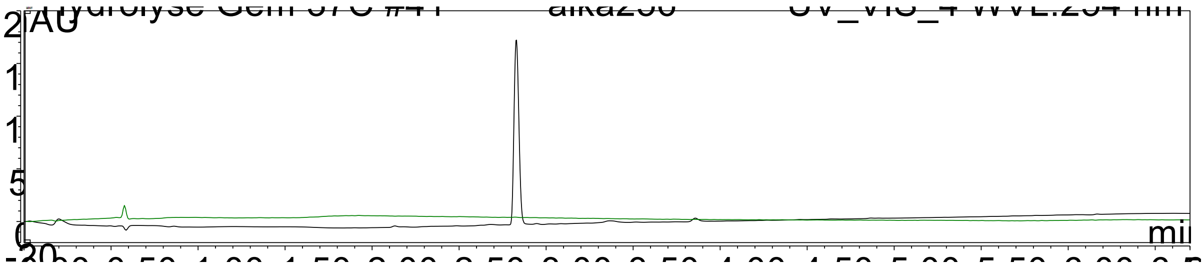

Figure S23: RP-HPLC chromatogram of CisPt-GemSucc-C_5Mal.

CarboPt-GemSucc-OAc

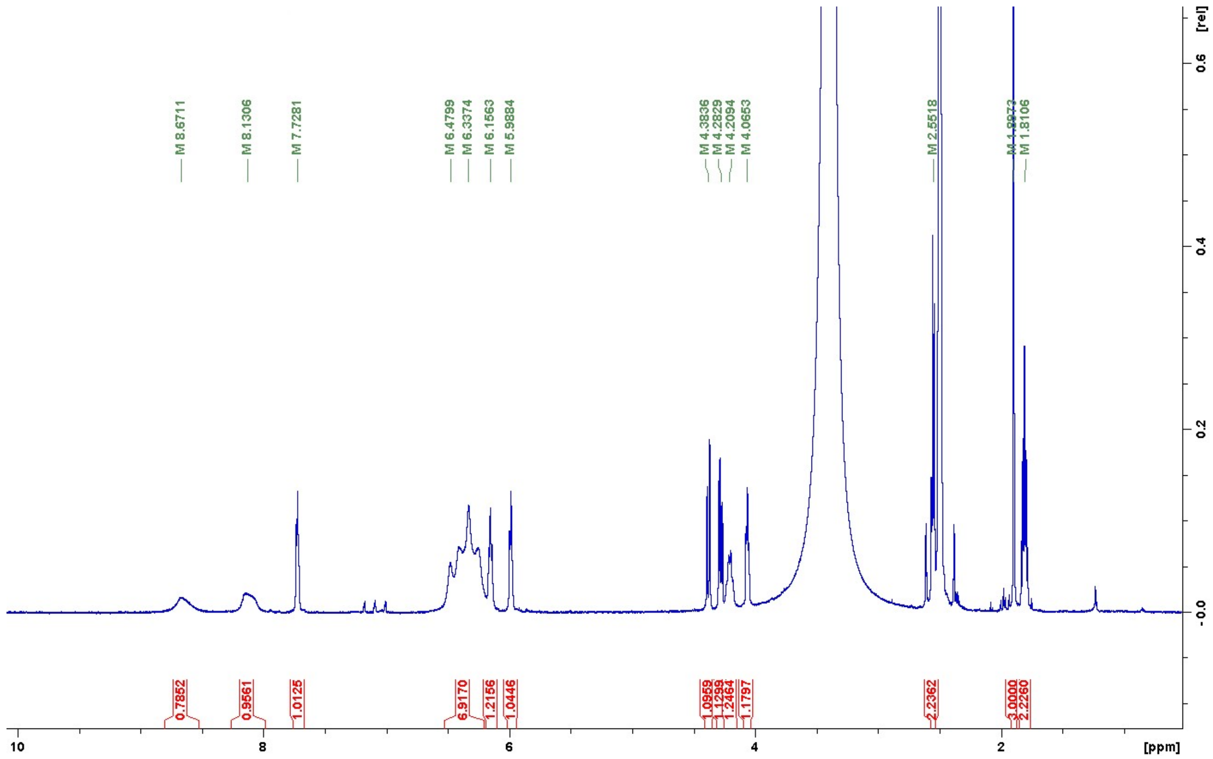

Figure S24: ^{1}H-NMR of CarboPt-GemSucc-OAc.

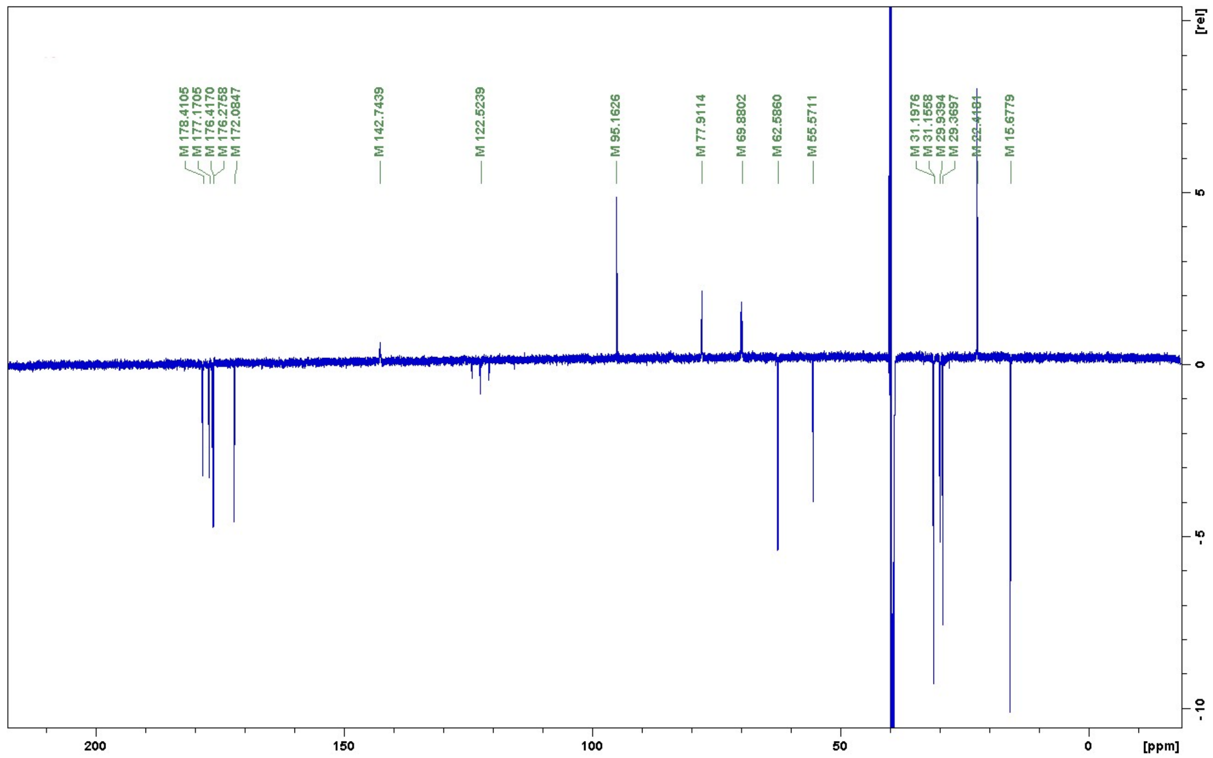

Figure S25: ^{13}C-NMR of CarboPt-GemSucc-OAc.

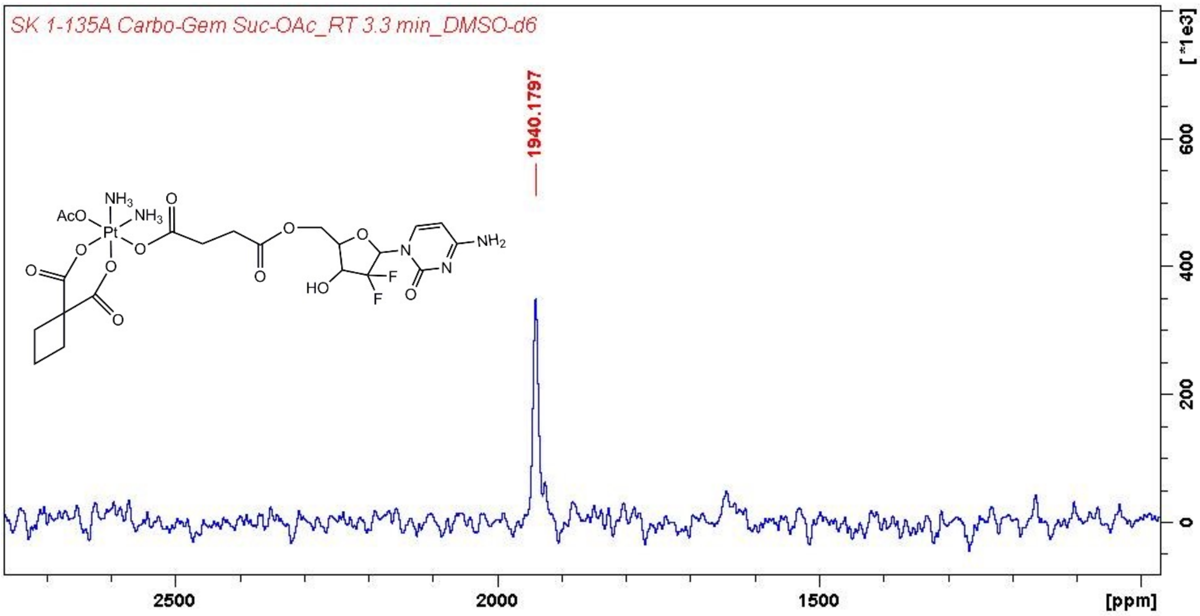

Figure S26: ^{195}Pt-NMR of CarboPt-GemSucc-OAc.

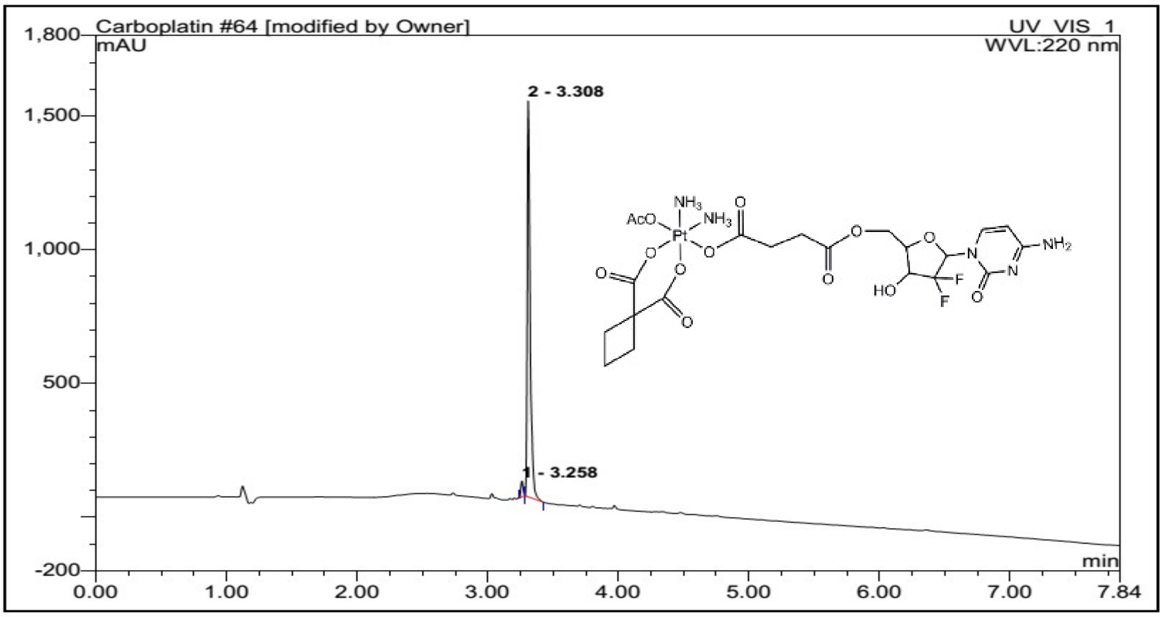

No.	Ret.Time min	Peak Name	Height mAU	Area mAU*min	Rel.Area %	Amount	Type
1	3.26	n.a.	59.692	1.132	2.89	n.a.	BMb*
2	3.31	n.a.	1480.308	38.094	97.11	n.a.	bMB*
Total:			1540.000	39.226	100.00	0.000	

Figure S27: RP-HPLC chromatogram of CarboPt-GemSucc-OAc.

CarboPt-GemSucc-C$_5$Mal

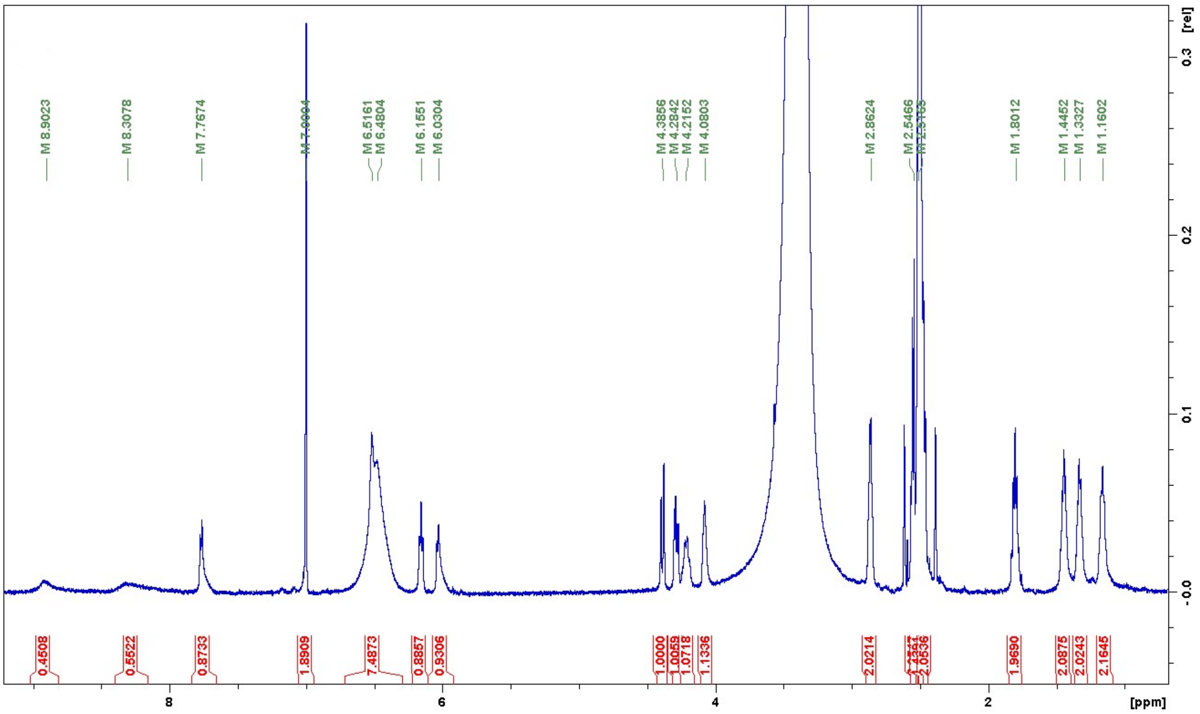

Figure S28: ^{1}H-NMR of CarboPt-GemSucc-C$_5$Mal.

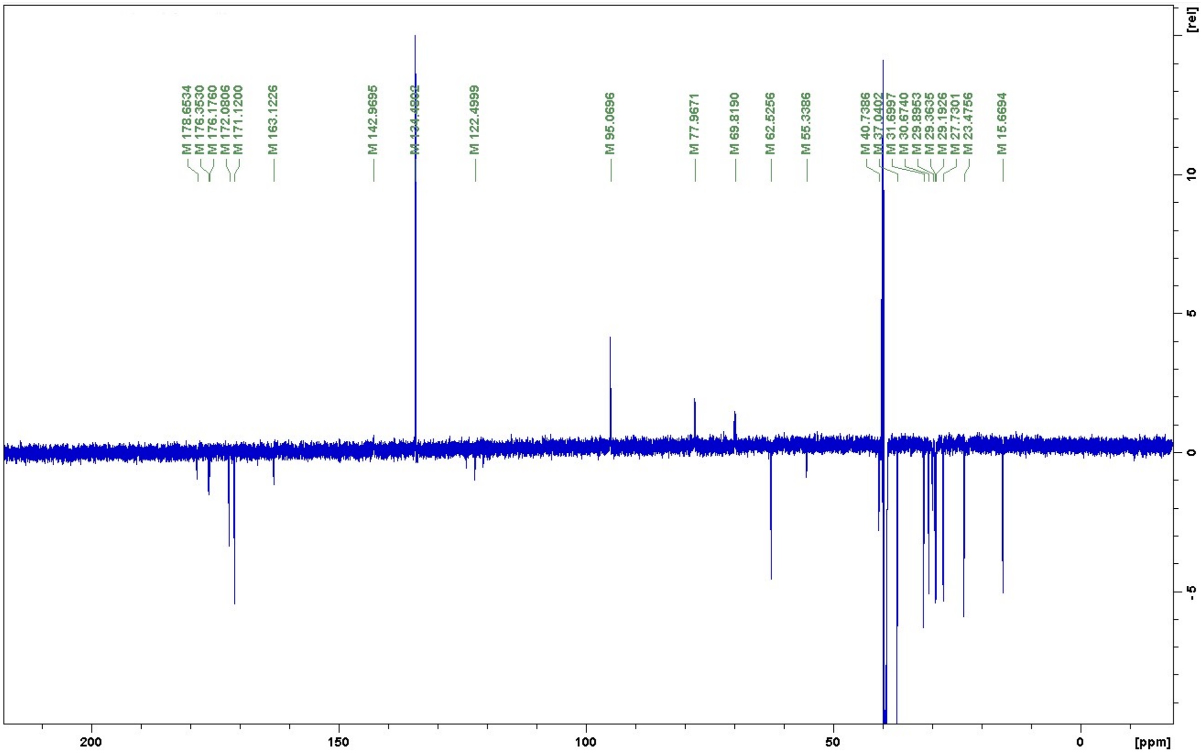

Figure S29: ^{13}C-NMR of CarboPt-GemSucc-C$_5$Mal.

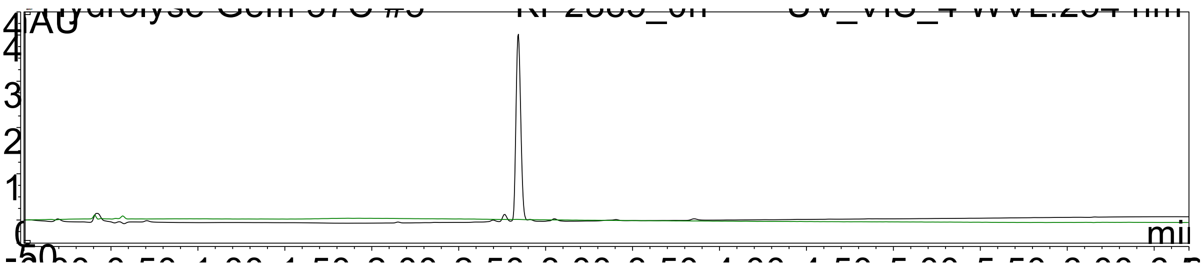

Figure S30: RP-HPLC chromatogram of CarboPt-GemSucc-C_5Mal.
